# Supplementary material for: Using Sex-Linked Markers via Genotyping-by-Sequencing to Identify XX/XY Sex Chromosomes in the Spiny Frog (Quasipaa boulengeri)
Source: Genes (Basel). 2022 Mar 24;13(4):575. doi: 10.3390/genes13040575 (PMC9027009; doi:10.3390/genes13040575)
Supplement: Supplementary file 1 [file genes-13-00575-s001.zip › genes-1596984-supplementary.pdf]

Supplementary Data S1

Specimen information and location data. Coordinates with three digital numbers after decimal points are GPS readings, and others are from maps. Specimen numbers with bold face were sequenced with GBS; specimen numbers in italic are specimens validated with electrophoresis; specimen numbers with underlined are specimens validated by DNA sequencing. Female individuals are represented by ♀ and male individuals are represented by ♂. For example, ***XM3690***♂ indicates that the male specimen has been sequenced with GBS and validated with electrophoresis. CIB, Chengdu Institute of Biology (Chengdu).

Quasipaa boulengeri (n=173)

1. Yan'e village, Dayi, Sichuan, China. N30°42.262, E103°26.710. *XM3664♀,* **XM3666♀, XM3667♀, XM3670♀, XM3673♀, XM3675♀, XM3679♀, XM3680♀, XM3682♀, XM3685♂, XM3686♂,** ***XM3690♂,*** **XM3691♂,** ***XM3692♂,*** *XM3712♂, XM3716♀,* **XM3750♀, XM3754♀, XM3757♀, XM3761♂, XM3764♂, XM3766♂, XM3770♂,** *XM3778♀, XM4115♀,* **XM4116♂, XM4117♂, XM4118♂, XM4120♂, XM4121♂, XM4122♂, XM4158♀, XM4159♂, XM4160♀, XM4161♂, XM4164♂, XM4166♀, XM4167♀, XM4168♀, XM4172♂,** *XM4174♂,* **XM4177♂,** *XM4178♂,* **XM4180♀, XM4213♀.** (DYYEC, 23♂; 22♀).

2. Heping village, Dayi, Sichuan, China. N30°43.172, E103°25.139. ***XM4201♀,*** **XM4202♂,** ***XM4203♀,*** **XM4205♀, XM4209♂.** (DYHPC, 2♂; 3♀).

3. Temple Gaotang, Dayi, Sichuan, China. N30°35.083, E103°27.949. XM3008♀, *XM3385♂, XM3630♂, XM3631♀, XM3633♂, XM3662♂, XM3733♀, XM3734♀, XM4240♂.* (DYGTS, 5♂; 4♀).

4. Heming township, Dayi, Sichuan, China. N30°36.103, E103°22.660. *XM4057♀, XM4058♂, XM4059♂, XM4063♀, XM4064♀.* (DYHM, 2♂; 3♀).

5. Wushan township, Dayi, Sichuan, China. N30°40.474, E103°23.448. *XM3736♀, XM3737♀, XM3738♂.* (DYWS, 1♂; 2♀).

6. Xiling town, Dayi, Sichuan, China. N30°66.013, E103°26.49. *XM3511♂, XM3517♀, XM3527♂, XM3528♀, XM3529♀.* (DYXL, 2♂; 3♀).

7. Xieyuan town, Dayi, Sichuan, China. N30°37.524, E103°22.218. *XM4087♂, XM4088♂, XM4089♀, XM4090♂, XM4091♀.* (DYXY, 3♂; 2♀).

8. Xinchang town, Dayi, Sichuan, China. N30°35.400, E103°21.944. *XM4107♀, XM4108♂, XM4109♀*. (DYXC, 1♂; 2♀).

9. Hongkou township, Dujiangyan, Sichuan, China. N31°120.45, E103°65.208. *XM4252♂, XM4253♂, XM4261♂, XM4262♂, XM4263♂*. (DJYHK, 5♂; 0♀).

10. Mt. Emei, Emeishan, Sichuan, China. N29°35.025, E103°17.301. *XM3371♀*. (EMS, 0♂; 1♀).

11. Luomu town, Emeishan, Sichuan, China. N29°30.945, E103°27.426. *XM3842♀, XM3843♂, XM3844♂, XM3845♀, XM3846♀*. (EMLM, 2♂; 3♀).

12. Puxing township, Emeishan, Sichuan, China. N29°41.497, E103°27.736. *XM3821♀, XM3799♀, XM3800♀, XM3804♀, XM3805♂, XM3806♂, XM4235♀*. (EMPX, 2♂; 5♀).

13. Liujiang town, Hongya, Sichuan, China. N29°43.3, E103°13.745. *XM3791♀, XM3827♀*. (HYLJ, 0♂; 2♀).

14. Huatou town, Jiajiang, Sichuan, China. N29°43.799, E103°23.438. *XM3824♀*. (JJHT, 0♂; 1♀).

15. Cifeng town, Pengzhou, Sichuan, China. N31°08.981, E103°43.630. *XM4283♂, XM4285♂, XM4288♂, XM4289♀, XM4291♀, XM4297♂, XM4298♀*. (PZCF, 4♂; 3♀).

16. Longmenshan town, Pengzhou, Sichuan, China. N31°14.630, E103°48.436. *XM4272♀, XM5752♂, XM5753♀, XM5754♂*. (PZLMS, 2♂; 2♀).

17. Mt. Qingcheng, Sichuan, China. N30°53.894, E103°30.818. *XM2934♀, XM2963♀,* <u>XM3075♀,</u> *XM3480♂, XM3481♂, XM3486♀, XM3492♀, XM3493♂, XM3494♀*. (QCS, 3♂; 6♀).

18. Datong township, Qionglai, Sichuan, China. N30°32.274, E103°17.989. *XM4039♂, XM4040♀, XM4041♂, XM4042♀, XM4043♂, XM4044♂, XM4045♀*. (QLDT, 4♂; 3♀).

19. Daozuo township, Qionglai, Sichuan, China. N30°18.405, E103°14.705. *XM3905♀, XM3906♀, XM3907♀, XM3908♂, XM3909♀, XM3910♂, XM3911♀*. (QLDZ, 2♂; 5♀).

20. Huojing town, Qionglai, Sichuan, China. N30°18.776, E103°13.009. *XM3944♀, XM3945♂, XM3946♀, XM3947♀*. (QLHJ, 1♂; 3♀).

21. Nanbao township, Qionglai, Sichuan, China. N30°25.678, E103°10.539. *XM4025♂, XM4026♀, XM4036♂*. (QLNB, 2♂; 1♀).

22. Mt. Tiantai, Qionglai, Sichuan, China. N30°15.396, E105°05.632. *QLY381♂, QLY384♂, XM3614♂, XM3616♂, XM3618♂, XM3620♀, XM3622♀, XM3623♀, XM3624♀, XM3626♀.* (QLTTS, 5♂; 5♀).

23. Shuikou town, Qionglai, Sichuan, China. N30°29.244, E103°13.260. *XM3948♂, XM3949♂, XM3958♂, XM3964M.* (QLTTS, 4♂; 0♀).

24. Xuankou town, Wenchuan, Sichuan, China. N30°57.860, E103°28.159. *XM3296♀, XM3743♀, XM3745♀, XM3746♀, XM3748♀*. (WCXK, 0♂; 5♀).

25. Yingxiu town, Wenchuan, Sichuan, China. N31°01.420, E103°23.819. *XM4239♀, XM4242♀, XM4369♀, XM4370♀, XM4371♂, XM4372♂*. (WCYX, 2♂; 4♀).

26. Bifeng canyon, Ya'an, Sichuan, China. N30°04.564, E102°59.374. *XM3570♀, XM3576♂, XM3577♀, XM3578♂, XM3579♂, XM3584M.* (YABFX, 4♂; 2♀).

Figure S1 The relationship between scaffold 1345 of *N. parkeri* and B relationship between chromosome 1 of *Q. boulengeri*.

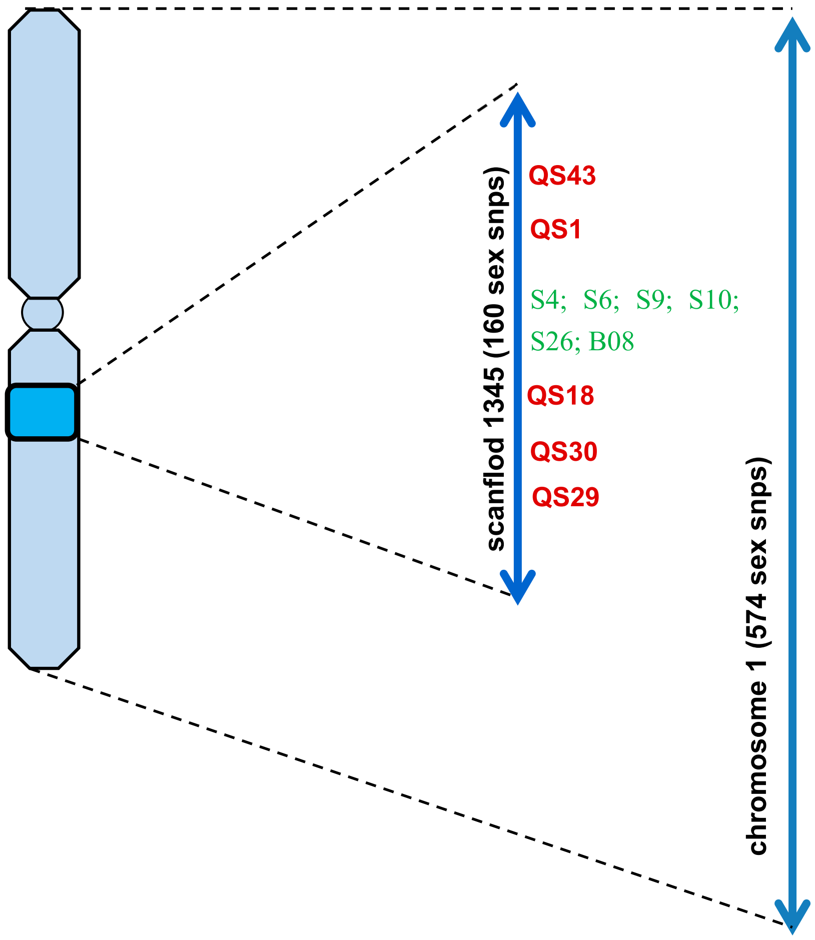

Note: All sex-linked microsatellites of *Q. boulengeri* (green front) were successfully compared to scaffold 1345 of *N. parkeri* [15, 21, 24], a closely related species of *Q. boulengeri*. Therefore, scaffold 1345 was homologous with chromosome 1 of *Q. boulengeri*. the confirmed sex-linked GBS-tags that located on the scaffold 1345 (green front) are more reliable to be chosen for PCR validation

Figure S2 PCR validation of 4 loci

Validation of the sex-linked SNP markers. The first primer design method was adopted for four SD sites, i.e., QS1, QS18, QS30, and QS43 (Table 3). After amplification in additional samples (10♀10♂) respectively from three populations. M and F followed the specimen number to indicate male and female. DNA sequencing showing that the sites with gender differences are in the red box, this sites appears as a double peak in males and a single peak in females.

QS1:

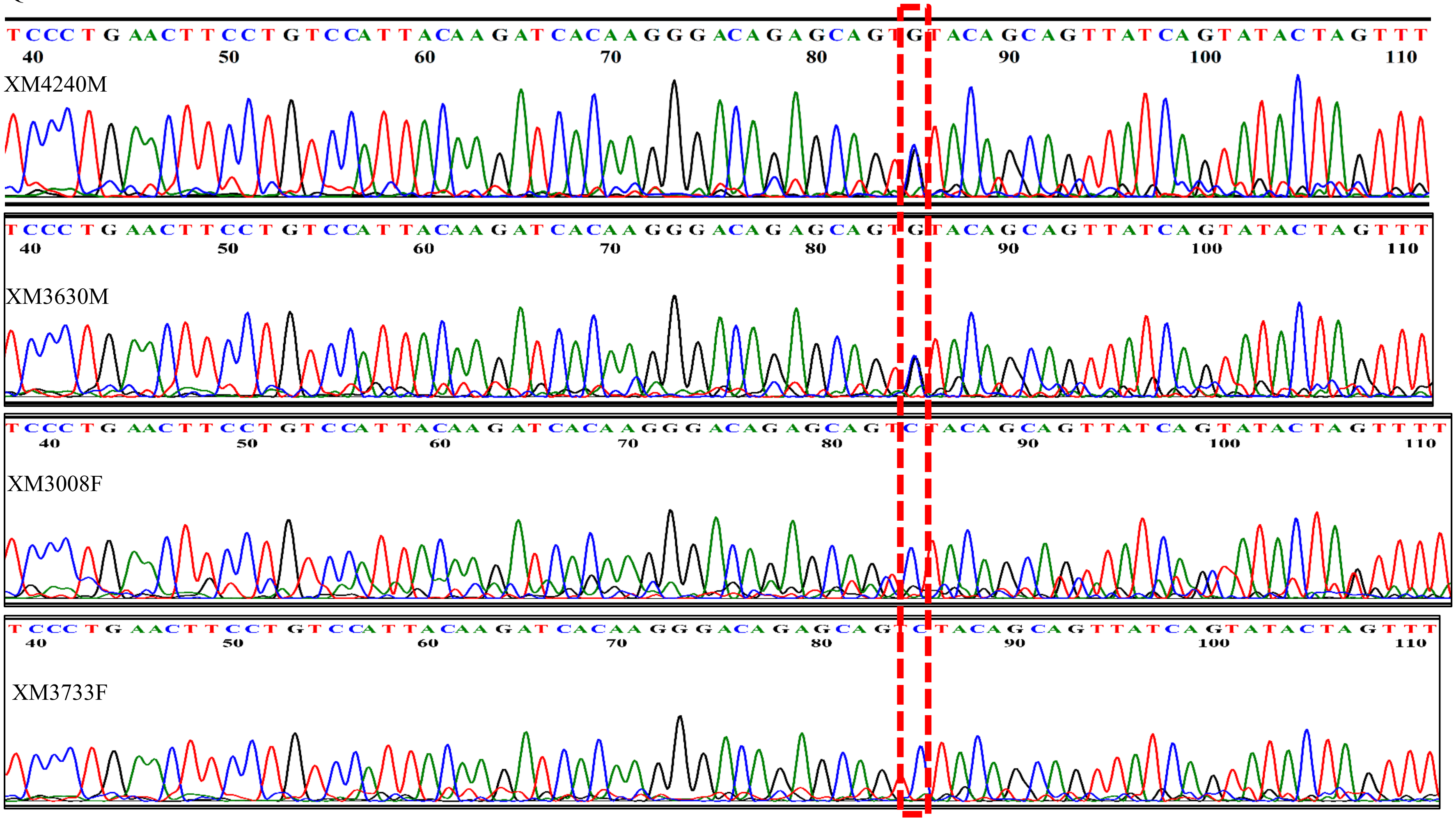

QS18:

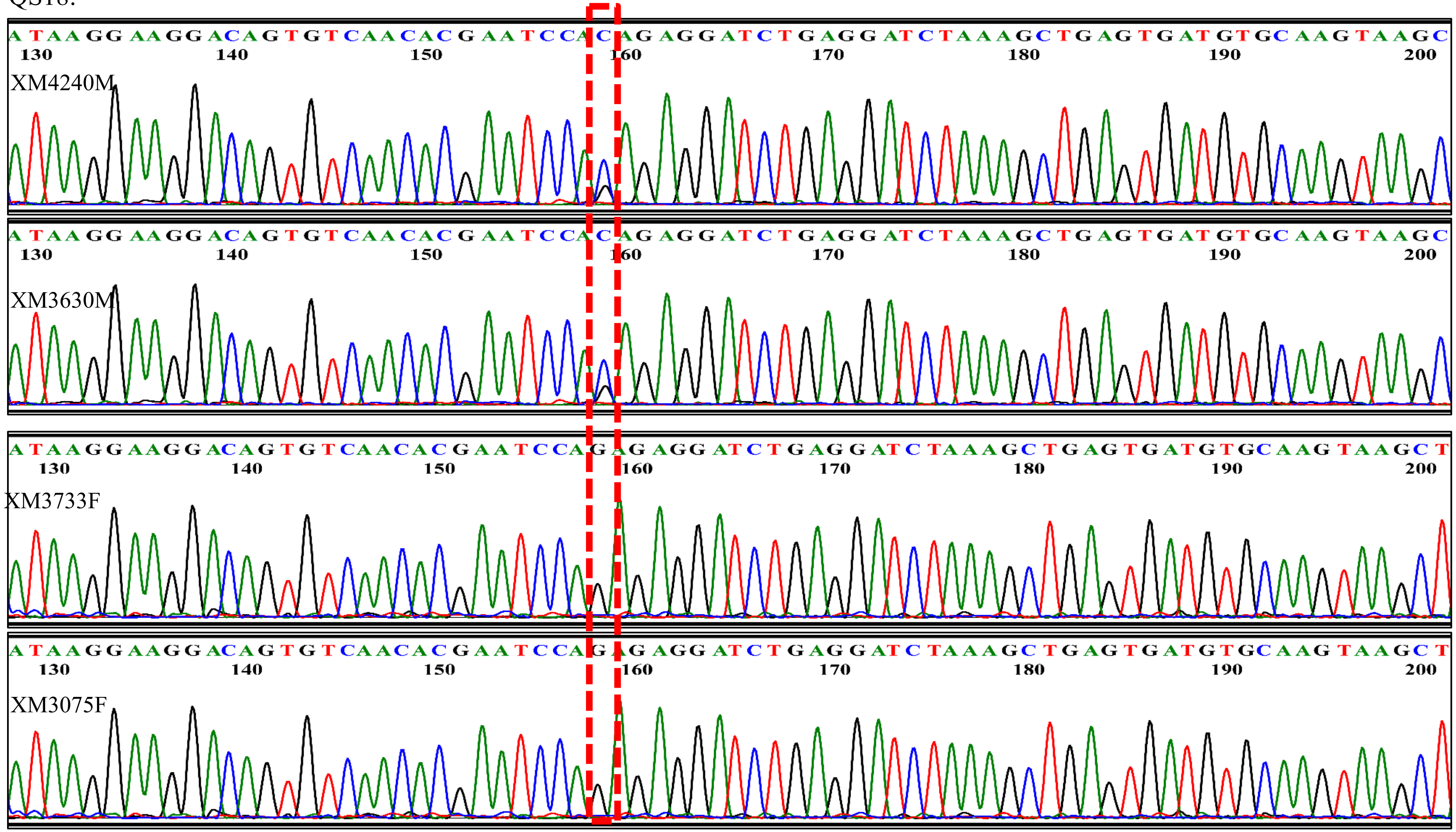

ATAAGGAAGGACAGTGTCAACACGAATCCACAGAGGATCTGAGGATCTAAAGCTGAGTGATGTGCAAGTAAGC
XM4240M
ATAAGGAAGGACAGTGTCAACACGAATCCACAGAGGATCTGAGGATCTAAAGCTGAGTGATGTGCAAGTAAGC
XM3630M
ATAAGGAAGGACAGTGTCAACACGAATCCAGAGAGGATCTGAGGATCTAAAGCTGAGTGATGTGCAAGTAAGCT
XM3733F
ATAAGGAAGGACAGTGTCAACACGAATCCAGAGAGGATCTGAGGATCTAAAGCTGAGTGATGTGCAAGTAAGCT
XM3075F

QS30:

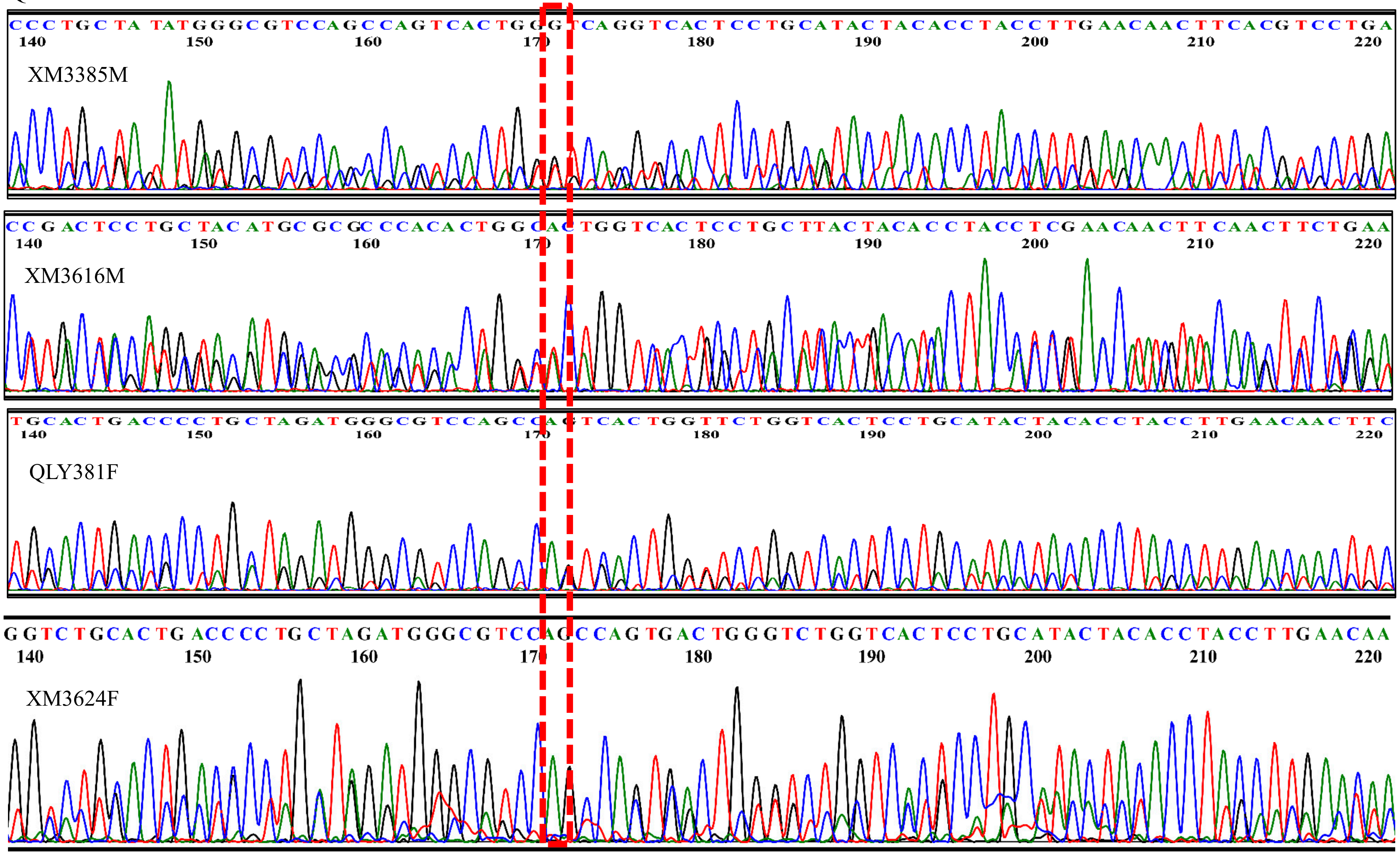

QS43:

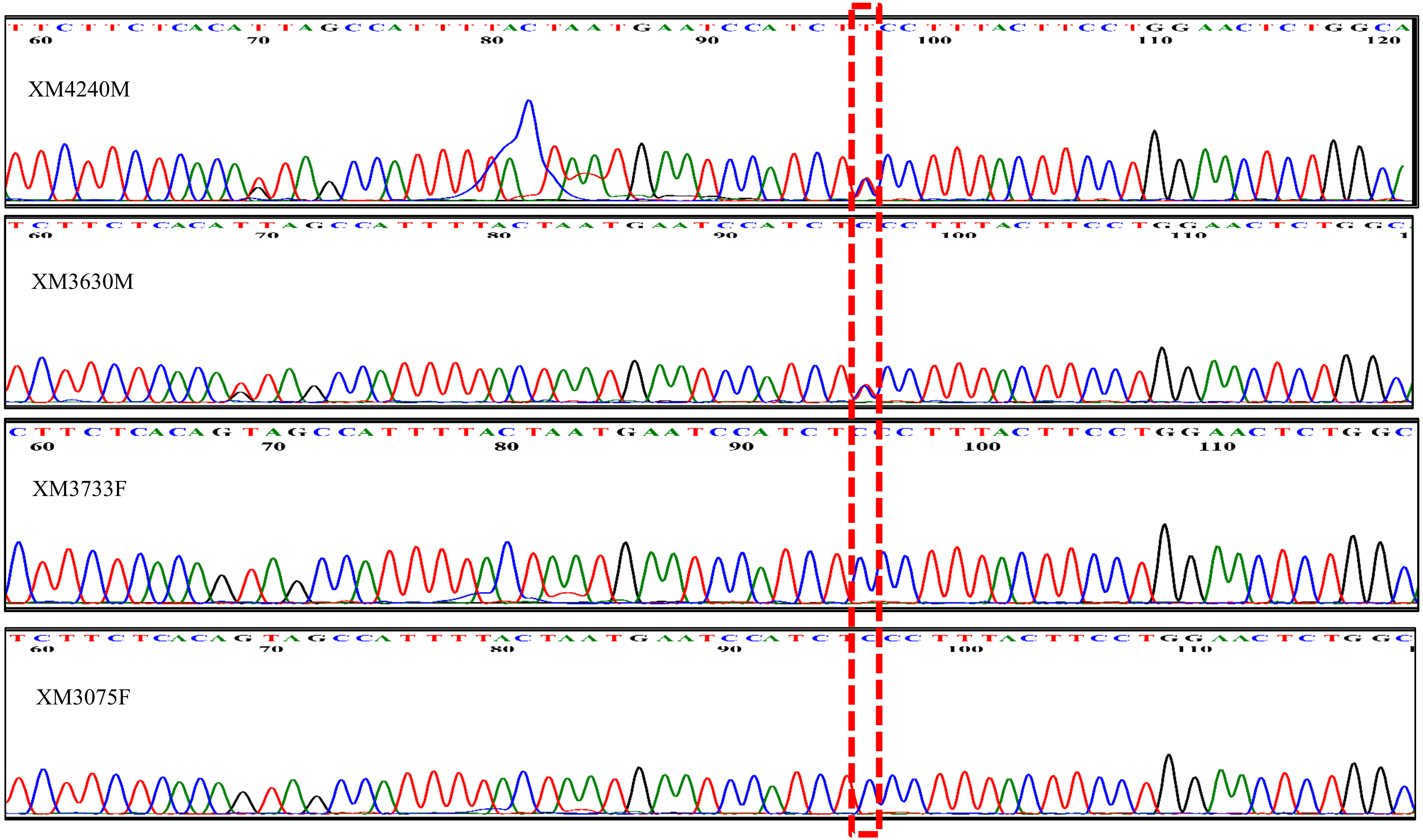

Table S1 Summary of sex-linked GBS tags

	Total Number	Frequency (XY)	Frequency (ZW)	Heterozygosity (XY)	Heterozygosity (ZW)	Y-limited	W-limited
Putatively	1049	2	0	122	0	905	21
Confirmed	581	2	0	49	0	523	7
PCR validation	5	-	-	4	-	-	-

Note: Summary of sex-linked GBS tags identified from three approaches. Frequency: Number of sex- linked markers identified from frequency differences approach; Heterozygosity: Number of sex-linked markers identified from heterozygosity differences; Y-limited: Number of sex-linked markers identified from male-limited occurrence approach; W-limited: Number of sex-linked markers identified from female-limited occurrence approach; Confirmed: Number of sex-linked markers mapped on *Q. boulengeri* chromosome 1; PCR validation: Number of sex-linked markers were verified by PCR.

Table S2 All details of sex-linked markers

Locus.ID (CLocus_)	Col	Pop.ID	P.Nuc	Q.Nuc	N	P	Obs.Het	Obs.Hom	Confirmed (Tor F)	System	approach
38828	28	F	G	-	21	1	0	1	T	XY	(i)frequency difference
38828	28	M	G	A	22	0.5	1	0			(i)frequency difference
38828	126	F	G	-	21	1	0	1			(i)frequency difference
38828	126	M	G	A	22	0.5	1	0			(i)frequency difference
614599	162	F	G	-	21	1	0	1	T	XY	(i)frequency difference
614599	162	M	G	T	22	0.4772727	0.95455	0.04545			(i)frequency difference
6020	94	F	C	-	12	1	0	1	T	XY	(ii)heterozygosity difference
6020	94	M	C	T	17	0.5	1	0			(ii)heterozygosity difference
7352	187	M	G	C	19	0.5	1	0	F		(ii)heterozygosity difference
7352	187	F	G	-	20	1	0	1			(ii)heterozygosity difference
9610	54	F	C	-	14	1	0	1	T	XY	(ii)heterozygosity difference
9610	54	M	C	T	19	0.5	1	0			(ii)heterozygosity difference
12357	5	F	A	-	15	1	0	1	F		(ii)heterozygosity difference
12357	5	M	A	C	21	0.5	1	0			(ii)heterozygosity difference

15844	213	F	C	-	15	1	0	1	F		(ii)heterozygosity difference
15844	74	F	G	-	16	1	0	1			(ii)heterozygosity difference
15844	189	F	G	-	16	1	0	1			(ii)heterozygosity difference
15844	268	F	A	-	16	1	0	1			(ii)heterozygosity difference
15844	74	M	G	A	21	0.5	1	0			(ii)heterozygosity difference
15844	189	M	G	C	21	0.5	1	0			(ii)heterozygosity difference
15844	213	M	C	A	21	0.5	1	0			(ii)heterozygosity difference
15844	268	M	A	T	21	0.5	1	0			(ii)heterozygosity difference
20265	15	F	T	-	19	1	0	1	F		(ii)heterozygosity difference
20265	217	F	G	-	19	1	0	1			(ii)heterozygosity difference
20265	217	M	G	A	19	0.5	1	0			(ii)heterozygosity difference
20265	280	F	C	-	19	1	0	1			(ii)heterozygosity difference
20265	280	M	C	T	19	0.5	1	0			(ii)heterozygosity difference
20265	15	M	T	C	20	0.5	1	0			(ii)heterozygosity difference
21104	282	F	C	-	15	1	0	1	T	XY	(ii)heterozygosity difference
21104	282	M	C	T	15	0.5	1	0			(ii)heterozygosity difference
21128	7	M	T	G	15	0.5	1	0	F		(ii)heterozygosity difference

21128	285	M	G	T	15	0.5	1	0			(ii)heterozygosity difference
21128	7	F	T	-	19	1	0	1			(ii)heterozygosity difference
21128	285	F	G	-	19	1	0	1			(ii)heterozygosity difference
21628	208	F	A	-	19	1	0	1	T	XY	(ii)heterozygosity difference
21628	208	M	A	G	19	0.5	1	0			(ii)heterozygosity difference
21628	241	F	G	-	19	1	0	1			(ii)heterozygosity difference
21628	241	M	G	A	19	0.5	1	0			(ii)heterozygosity difference
22128	238	F	G	-	13	1	0	1	F		(ii)heterozygosity difference
22128	238	M	G	A	15	0.5	1	0			(ii)heterozygosity difference
24490	229	F	G	-	16	1	0	1	T	XY	(ii)heterozygosity difference
24490	87	F	C	-	20	1	0	1			(ii)heterozygosity difference
24490	87	M	C	T	21	0.5	1	0			(ii)heterozygosity difference
24490	229	M	G	A	21	0.5	1	0			(ii)heterozygosity difference
26133	290	M	T	G	17	0.5	1	0	F		(ii)heterozygosity difference
26133	296	M	T	A	17	0.5	1	0			(ii)heterozygosity difference
26133	290	F	T	-	18	1	0	1			(ii)heterozygosity difference
26133	296	F	T	-	18	1	0	1			(ii)heterozygosity difference

27723	170	M	C	T	16	0.5	1	0	F		(ii)heterozygosity difference
27723	170	F	C	-	17	1	0	1			(ii)heterozygosity difference
31523	197	M	C	G	13	0.5	1	0	F		(ii)heterozygosity difference
31523	197	F	C	-	15	1	0	1			(ii)heterozygosity difference
32556	3	M	T	G	14	0.5	1	0	F		(ii)heterozygosity difference
32556	3	F	T	-	20	1	0	1			(ii)heterozygosity difference
32964	238	F	A	-	16	1	0	1	F		(ii)heterozygosity difference
32964	238	M	A	C	17	0.5	1	0			(ii)heterozygosity difference
34389	235	F	C	-	20	1	0	1	T	XY	(ii)heterozygosity difference
34389	235	M	C	T	21	0.5	1	0			(ii)heterozygosity difference
35863	175	F	A	-	18	1	0	1	T	XY	(ii)heterozygosity difference
35863	175	M	A	T	18	0.5	1	0			(ii)heterozygosity difference
35863	19	M	G	A	19	0.5	1	0			(ii)heterozygosity difference
35863	77	M	A	T	19	0.5	1	0			(ii)heterozygosity difference
35863	19	F	G	-	20	1	0	1			(ii)heterozygosity difference
35863	77	F	A	-	20	1	0	1			(ii)heterozygosity difference
38828	159	M	C	G	19	0.5	1	0	T	XY	(ii)heterozygosity difference

38828	28	F	G	-	21	1	0	1			(ii)heterozygosity difference
38828	126	F	G	-	21	1	0	1			(ii)heterozygosity difference
38828	159	F	C	-	21	1	0	1			(ii)heterozygosity difference
38828	28	M	G	A	22	0.5	1	0			(ii)heterozygosity difference
38828	126	M	G	A	22	0.5	1	0			(ii)heterozygosity difference
45859	190	F	T	-	14	1	0	1	F		(ii)heterozygosity difference
45859	190	M	T	A	17	0.5	1	0			(ii)heterozygosity difference
52751	157	F	T	-	15	1	0	1	F		(ii)heterozygosity difference
52751	178	F	A	-	15	1	0	1			(ii)heterozygosity difference
52751	188	F	G	-	15	1	0	1			(ii)heterozygosity difference
52751	157	M	T	A	19	0.5	1	0			(ii)heterozygosity difference
52751	178	M	A	C	19	0.5	1	0			(ii)heterozygosity difference
52751	188	M	G	A	19	0.5	1	0			(ii)heterozygosity difference
52943	51	F	G	-	20	1	0	1	T	XY	(ii)heterozygosity difference
52943	51	M	G	A	20	0.5	1	0			(ii)heterozygosity difference
52943	183	F	G	-	20	1	0	1			(ii)heterozygosity difference
52943	183	M	G	A	20	0.5	1	0			(ii)heterozygosity difference

52943	261	F	T	-	20	1	0	1			(ii)heterozygosity difference
52943	261	M	T	C	20	0.5	1	0			(ii)heterozygosity difference
61677	37	M	G	A	17	0.5	1	0	T	XY	(ii)heterozygosity difference
61677	6	M	G	C	18	0.5	1	0			(ii)heterozygosity difference
61677	41	M	C	G	18	0.5	1	0			(ii)heterozygosity difference
61677	267	M	A	T	18	0.5	1	0			(ii)heterozygosity difference
61677	289	M	A	G	18	0.5	1	0			(ii)heterozygosity difference
61677	6	F	G	-	20	1	0	1			(ii)heterozygosity difference
61677	37	F	G	-	20	1	0	1			(ii)heterozygosity difference
61677	41	F	C	-	20	1	0	1			(ii)heterozygosity difference
61677	267	F	A	-	20	1	0	1			(ii)heterozygosity difference
61677	289	F	A	-	20	1	0	1			(ii)heterozygosity difference
63317	124	M	G	C	15	0.5	1	0	T	XY	(ii)heterozygosity difference
63317	141	M	G	C	15	0.5	1	0			(ii)heterozygosity difference
63317	124	F	G	-	20	1	0	1			(ii)heterozygosity difference
63317	141	F	G	-	20	1	0	1			(ii)heterozygosity difference
70112	55	F	C	-	16	1	0	1	T	XY	(ii)heterozygosity difference

70112	55	M	C	G	16	0.5	1	0			(ii)heterozygosity difference
73728	174	F	G	-	12	1	0	1	T	XY	(ii)heterozygosity difference
73728	174	M	G	A	12	0.5	1	0			(ii)heterozygosity difference
74283	101	M	C	G	16	0.5	1	0	T	XY	(ii)heterozygosity difference
74283	101	F	C	-	17	1	0	1			(ii)heterozygosity difference
75198	284	M	A	G	12	0.5	1	0	T	XY	(ii)heterozygosity difference
75198	284	F	A	-	17	1	0	1			(ii)heterozygosity difference
75421	217	M	G	C	17	0.5	1	0	F		(ii)heterozygosity difference
75421	117	M	C	T	18	0.5	1	0			(ii)heterozygosity difference
75421	117	F	C	-	20	1	0	1			(ii)heterozygosity difference
75421	217	F	G	-	20	1	0	1			(ii)heterozygosity difference
79085	18	F	C	-	14	1	0	1	F		(ii)heterozygosity difference
79085	18	M	C	T	15	0.5	1	0			(ii)heterozygosity difference
80479	37	F	C	-	14	1	0	1	T	XY	(ii)heterozygosity difference
80479	193	F	G	-	14	1	0	1			(ii)heterozygosity difference
80479	259	F	G	-	14	1	0	1			(ii)heterozygosity difference
80479	37	M	C	T	16	0.5	1	0			(ii)heterozygosity difference

80479	193	M	G	A	16	0.5	1	0			(ii)heterozygosity difference
80479	259	M	G	T	16	0.5	1	0			(ii)heterozygosity difference
88297	99	M	G	C	18	0.5	1	0	F		(ii)heterozygosity difference
88297	107	M	G	T	18	0.5	1	0			(ii)heterozygosity difference
88297	99	F	G	-	20	1	0	1			(ii)heterozygosity difference
88297	107	F	G	-	20	1	0	1			(ii)heterozygosity difference
90811	231	F	C	-	12	1	0	1	F		(ii)heterozygosity difference
90811	231	M	C	T	13	0.5	1	0			(ii)heterozygosity difference
100202	135	F	C	-	19	1	0	1	T	XY	(ii)heterozygosity difference
100202	135	M	C	A	22	0.5	1	0			(ii)heterozygosity difference
114862	273	F	A	-	16	1	0	1	F		(ii)heterozygosity difference
114862	273	M	A	G	16	0.5	1	0			(ii)heterozygosity difference
153573	185	M	G	C	14	0.5	1	0	F		(ii)heterozygosity difference
153573	185	F	G	-	16	1	0	1			(ii)heterozygosity difference
163635	20	F	C	-	13	1	0	1	T	XY	(ii)heterozygosity difference
163635	76	F	G	-	13	1	0	1			(ii)heterozygosity difference
163635	90	F	A	-	13	1	0	1			(ii)heterozygosity difference

163635	126	F	C	-	13	1	0	1			(ii)heterozygosity difference
163635	158	F	T	-	13	1	0	1			(ii)heterozygosity difference
163635	203	F	G	-	13	1	0	1			(ii)heterozygosity difference
163635	282	F	C	-	13	1	0	1			(ii)heterozygosity difference
163635	159	M	A	T	14	0.5	1	0			(ii)heterozygosity difference
163635	159	F	A	-	15	1	0	1			(ii)heterozygosity difference
163635	76	M	G	A	16	0.5	1	0			(ii)heterozygosity difference
163635	203	M	G	A	16	0.5	1	0			(ii)heterozygosity difference
163635	20	M	C	T	17	0.5	1	0			(ii)heterozygosity difference
163635	90	M	A	T	17	0.5	1	0			(ii)heterozygosity difference
163635	126	M	C	T	17	0.5	1	0			(ii)heterozygosity difference
163635	158	M	T	G	17	0.5	1	0			(ii)heterozygosity difference
163635	282	M	C	T	17	0.5	1	0			(ii)heterozygosity difference
165389	29	F	T	-	14	1	0	1	F		(ii)heterozygosity difference
165389	29	M	T	A	15	0.5	1	0			(ii)heterozygosity difference
191748	65	F	A	-	12	1	0	1	F		(ii)heterozygosity difference
191748	65	M	A	C	12	0.5	1	0			(ii)heterozygosity difference

192925	75	M	C	T	13	0.5	1	0	F		(ii)heterozygosity difference
192925	197	M	T	A	13	0.5	1	0			(ii)heterozygosity difference
192925	75	F	C	-	15	1	0	1			(ii)heterozygosity difference
192925	197	F	T	-	15	1	0	1			(ii)heterozygosity difference
196323	93	M	G	A	13	0.5	1	0	T	XY	(ii)heterozygosity difference
196323	93	F	G	-	15	1	0	1			(ii)heterozygosity difference
199491	212	M	G	A	16	0.5	1	0	T	XY	(ii)heterozygosity difference
199491	77	M	C	T	17	0.5	1	0			(ii)heterozygosity difference
199491	245	M	G	T	17	0.5	1	0			(ii)heterozygosity difference
199491	77	F	C	-	20	1	0	1			(ii)heterozygosity difference
199491	212	F	G	-	20	1	0	1			(ii)heterozygosity difference
199491	245	F	G	-	20	1	0	1			(ii)heterozygosity difference
226924	5	F	C	-	15	1	0	1	T	XY	(ii)heterozygosity difference
226924	5	M	C	A	20	0.5	1	0			(ii)heterozygosity difference
242475	212	F	C	-	13	1	0	1	F		(ii)heterozygosity difference
242475	212	M	C	T	17	0.5	1	0			(ii)heterozygosity difference
243881	60	M	A	G	20	0.5	1	0	F		(ii)heterozygosity difference

243881	111	M	C	G	20	0.5	1	0			(ii)heterozygosity difference
243881	60	F	A	-	21	1	0	1			(ii)heterozygosity difference
243881	111	F	C	-	21	1	0	1			(ii)heterozygosity difference
255076	60	F	T	-	20	1	0	1	F		(ii)heterozygosity difference
255076	60	M	T	G	20	0.5	1	0			(ii)heterozygosity difference
263452	37	M	C	T	12	0.5	1	0	T	XY	(ii)heterozygosity difference
263452	86	M	T	C	12	0.5	1	0			(ii)heterozygosity difference
263452	86	F	T	-	17	1	0	1			(ii)heterozygosity difference
263452	37	F	C	-	18	1	0	1			(ii)heterozygosity difference
280937	213	M	T	G	12	0.5	1	0	F		(ii)heterozygosity difference
280937	213	F	T	-	13	1	0	1			(ii)heterozygosity difference
285497	195	M	G	A	19	0.5	1	0	T	XY	(ii)heterozygosity difference
285497	195	F	G	-	20	1	0	1			(ii)heterozygosity difference
310734	11	M	T	A	15	0.5	1	0	F		(ii)heterozygosity difference
310734	11	F	T	-	17	1	0	1			(ii)heterozygosity difference
313811	200	F	C	-	16	1	0	1	T	XY	(ii)heterozygosity difference
313811	200	M	C	A	16	0.5	1	0			(ii)heterozygosity difference

317631	169	M	G	A	18	0.5	1	0	F		(ii)heterozygosity difference
317631	169	F	G	-	20	1	0	1			(ii)heterozygosity difference
327357	137	M	G	A	15	0.5	1	0	F		(ii)heterozygosity difference
327357	167	M	G	T	15	0.5	1	0			(ii)heterozygosity difference
327357	137	F	G	-	17	1	0	1			(ii)heterozygosity difference
327357	167	F	G	-	17	1	0	1			(ii)heterozygosity difference
335383	23	F	T	-	18	1	0	1	F		(ii)heterozygosity difference
335383	23	M	T	C	18	0.5	1	0			(ii)heterozygosity difference
336017	201	M	C	T	16	0.5	1	0	F		(ii)heterozygosity difference
336017	201	F	C	-	17	1	0	1			(ii)heterozygosity difference
343561	236	F	G	-	16	1	0	1	F		(ii)heterozygosity difference
343561	236	M	G	A	19	0.5	1	0			(ii)heterozygosity difference
351465	75	F	C	-	13	1	0	1	F		(ii)heterozygosity difference
351465	75	M	C	T	13	0.5	1	0			(ii)heterozygosity difference
367607	41	M	G	T	16	0.5	1	0	F		(ii)heterozygosity difference
367607	172	M	T	C	16	0.5	1	0			(ii)heterozygosity difference
367607	41	F	G	-	18	1	0	1			(ii)heterozygosity difference

367607	172	F	T	-	18	1	0	1			(ii)heterozygosity difference
368395	50	F	A	-	17	1	0	1	F		(ii)heterozygosity difference
368395	50	M	A	G	17	0.5	1	0			(ii)heterozygosity difference
368395	51	F	A	-	17	1	0	1			(ii)heterozygosity difference
368395	51	M	A	C	17	0.5	1	0			(ii)heterozygosity difference
371440	253	F	C	-	17	1	0	1	T	XY	(ii)heterozygosity difference
371440	253	M	C	T	17	0.5	1	0			(ii)heterozygosity difference
377865	168	M	G	C	16	0.5	1	0	T	XY	(ii)heterozygosity difference
377865	207	M	T	G	16	0.5	1	0			(ii)heterozygosity difference
377865	168	F	G	-	18	1	0	1			(ii)heterozygosity difference
377865	207	F	T	-	18	1	0	1			(ii)heterozygosity difference
386683	8	F	C	-	12	1	0	1	T	XY	(ii)heterozygosity difference
386683	12	F	C	-	12	1	0	1			(ii)heterozygosity difference
386683	174	F	G	-	12	1	0	1			(ii)heterozygosity difference
386683	8	M	C	T	14	0.5	1	0			(ii)heterozygosity difference
386683	12	M	C	A	14	0.5	1	0			(ii)heterozygosity difference
386683	174	M	G	A	14	0.5	1	0			(ii)heterozygosity difference

389817	93	M	C	G	17	0.5	1	0	F		(ii)heterozygosity difference
389817	111	M	C	T	17	0.5	1	0			(ii)heterozygosity difference
389817	93	F	C	-	19	1	0	1			(ii)heterozygosity difference
389817	111	F	C	-	19	1	0	1			(ii)heterozygosity difference
446613	170	F	G	-	16	1	0	1	F		(ii)heterozygosity difference
446613	170	M	G	A	17	0.5	1	0			(ii)heterozygosity difference
448998	83	M	G	A	13	0.5	1	0	T	XY	(ii)heterozygosity difference
448998	83	F	G	-	14	1	0	1			(ii)heterozygosity difference
454707	155	M	A	G	13	0.5	1	0	T	XY	(ii)heterozygosity difference
454707	155	F	A	-	17	1	0	1			(ii)heterozygosity difference
455017	226	M	C	T	17	0.5	1	0			(ii)heterozygosity difference
455017	85	F	T	-	18	1	0	1			(ii)heterozygosity difference
455017	85	M	T	C	18	0.5	1	0			(ii)heterozygosity difference
455017	102	F	T	-	18	1	0	1			(ii)heterozygosity difference
455017	158	F	G	-	18	1	0	1			(ii)heterozygosity difference
455017	158	M	G	A	18	0.5	1	0			(ii)heterozygosity difference
455017	166	F	T	-	18	1	0	1			(ii)heterozygosity difference

455017	172	F	T	-	18	1	0	1			(ii)heterozygosity difference
455017	226	F	C	-	18	1	0	1			(ii)heterozygosity difference
455017	242	F	T	-	18	1	0	1			(ii)heterozygosity difference
455017	279	F	G	-	18	1	0	1			(ii)heterozygosity difference
455017	102	M	T	C	19	0.5	1	0			(ii)heterozygosity difference
455017	166	M	T	A	19	0.5	1	0			(ii)heterozygosity difference
455017	172	M	T	C	19	0.5	1	0			(ii)heterozygosity difference
455017	242	M	T	A	19	0.5	1	0			(ii)heterozygosity difference
455017	279	M	G	C	19	0.5	1	0			(ii)heterozygosity difference
465977	51	F	C	-	15	1	0	1	T	XY	(ii)heterozygosity difference
465977	93	F	T	-	15	1	0	1			(ii)heterozygosity difference
465977	106	F	G	-	15	1	0	1			(ii)heterozygosity difference
465977	133	F	C	-	15	1	0	1			(ii)heterozygosity difference
465977	180	F	T	-	15	1	0	1			(ii)heterozygosity difference
465977	204	F	A	-	15	1	0	1			(ii)heterozygosity difference
465977	51	M	C	T	17	0.5	1	0			(ii)heterozygosity difference
465977	93	M	T	C	17	0.5	1	0			(ii)heterozygosity difference

465977	106	M	G	T	17	0.5	1	0			(ii)heterozygosity difference
465977	133	M	C	T	17	0.5	1	0			(ii)heterozygosity difference
465977	180	M	T	A	17	0.5	1	0			(ii)heterozygosity difference
465977	204	M	A	G	17	0.5	1	0			(ii)heterozygosity difference
470004	200	F	G	-	12	1	0	1	F		(ii)heterozygosity difference
470004	200	M	G	A	18	0.5	1	0			(ii)heterozygosity difference
482347	131	F	A	-	12	1	0	1	F		(ii)heterozygosity difference
482347	131	M	A	C	20	0.5	1	0			(ii)heterozygosity difference
496473	40	F	A	-	13	1	0	1	F		(ii)heterozygosity difference
496473	40	M	A	T	13	0.5	1	0			(ii)heterozygosity difference
503155	186	F	G	-	17	1	0	1	F		(ii)heterozygosity difference
503155	186	M	G	T	21	0.5	1	0			(ii)heterozygosity difference
506937	280	M	G	A	15	0.5	1	0	F		(ii)heterozygosity difference
506937	280	F	G	-	17	1	0	1			(ii)heterozygosity difference
517207	94	M	C	G	14	0.5	1	0	F		(ii)heterozygosity difference
517207	94	F	C	-	15	1	0	1			(ii)heterozygosity difference
533737	267	F	A	-	13	1	0	1	F		(ii)heterozygosity difference

533737	267	M	A	T	14	0.5	1	0			(ii)heterozygosity difference
566624	115	F	G	-	17	1	0	1	T	XY	(ii)heterozygosity difference
566624	115	M	G	A	17	0.5	1	0			(ii)heterozygosity difference
580055	159	F	C	-	12	1	0	1	F		(ii)heterozygosity difference
580055	159	M	C	T	18	0.5	1	0			(ii)heterozygosity difference
625549	104	M	T	C	18	0.5	1	0	T	XY	(ii)heterozygosity difference
625549	71	M	C	T	19	0.5	1	0			(ii)heterozygosity difference
625549	71	F	C	-	20	1	0	1			(ii)heterozygosity difference
625549	104	F	T	-	20	1	0	1			(ii)heterozygosity difference
642057	64	F	T	-	13	1	0	1	T	XY	(ii)heterozygosity difference
642057	64	M	T	G	18	0.5	1	0			(ii)heterozygosity difference
642128	242	F	A	-	12	1	0	1	F		(ii)heterozygosity difference
642128	242	M	A	C	20	0.5	1	0			(ii)heterozygosity difference
654294	241	M	C	A	14	0.5	1	0	F		(ii)heterozygosity difference
654294	241	F	C	-	17	1	0	1			(ii)heterozygosity difference
663174	177	M	C	T	13	0.5	1	0	F		(ii)heterozygosity difference
663174	177	F	C	-	14	1	0	1			(ii)heterozygosity difference

668667	100	M	A	T	13	0.5	1	0	F		(ii)heterozygosity difference
668667	100	F	A	-	17	1	0	1			(ii)heterozygosity difference
668667	200	F	G	-	17	1	0	1			(ii)heterozygosity difference
668667	200	M	G	C	17	0.5	1	0			(ii)heterozygosity difference
679345	108	F	A	-	16	1	0	1	F		(ii)heterozygosity difference
679345	108	M	A	G	16	0.5	1	0			(ii)heterozygosity difference
679345	210	F	T	-	16	1	0	1			(ii)heterozygosity difference
679345	210	M	T	A	16	0.5	1	0			(ii)heterozygosity difference
687598	292	F	T	-	16	1	0	1	F		(ii)heterozygosity difference
687598	292	M	T	G	17	0.5	1	0			(ii)heterozygosity difference
707760	30	F	G	-	15	1	0	1	F		(ii)heterozygosity difference
707760	30	M	G	C	18	0.5	1	0			(ii)heterozygosity difference
712545	122	M	C	G	12	0.5	1	0	T	XY	(ii)heterozygosity difference
712545	122	F	C	-	14	1	0	1			(ii)heterozygosity difference
713062	8	F	G	-	19	1	0	1	F		(ii)heterozygosity difference
713062	97	F	T	-	19	1	0	1			(ii)heterozygosity difference
713062	97	M	T	C	19	0.5	1	0			(ii)heterozygosity difference

713062	8	M	G	C	21	0.5	1	0			(ii)heterozygosity difference
718951	52	F	G	-	12	1	0	1	T	XY	(ii)heterozygosity difference
718951	52	M	G	A	12	0.5	1	0			(ii)heterozygosity difference
723351	135	M	C	T	17	0.5	1	0	F		(ii)heterozygosity difference
723351	5	F	T	-	18	1	0	1			(ii)heterozygosity difference
723351	5	M	T	C	18	0.5	1	0			(ii)heterozygosity difference
723351	135	F	C	-	18	1	0	1			(ii)heterozygosity difference
723351	224	F	G	-	18	1	0	1			(ii)heterozygosity difference
723351	224	M	G	C	18	0.5	1	0			(ii)heterozygosity difference
723351	287	F	A	-	18	1	0	1			(ii)heterozygosity difference
723351	287	M	A	C	18	0.5	1	0			(ii)heterozygosity difference
723351	292	F	A	-	18	1	0	1			(ii)heterozygosity difference
723351	292	M	A	C	18	0.5	1	0			(ii)heterozygosity difference
738932	113	M	C	A	12	0.5	1	0	T	XY	(ii)heterozygosity difference
738932	173	M	C	T	12	0.5	1	0			(ii)heterozygosity difference
738932	228	M	C	T	12	0.5	1	0			(ii)heterozygosity difference
738932	113	F	C	-	14	1	0	1			(ii)heterozygosity difference

738932	173	F	C	-	14	1	0	1			(ii)heterozygosity difference
738932	228	F	C	-	14	1	0	1			(ii)heterozygosity difference
745464	196	F	G	-	17	1	0	1	T	XY	(ii)heterozygosity difference
745464	196	M	G	A	17	0.5	1	0			(ii)heterozygosity difference
745464	218	F	G	-	17	1	0	1			(ii)heterozygosity difference
745464	218	M	G	C	17	0.5	1	0			(ii)heterozygosity difference
745464	232	F	T	-	17	1	0	1			(ii)heterozygosity difference
745464	232	M	T	C	17	0.5	1	0			(ii)heterozygosity difference
745767	31	M	C	A	14	0.5	1	0	F		(ii)heterozygosity difference
745767	47	M	A	G	14	0.5	1	0			(ii)heterozygosity difference
745767	88	M	A	G	14	0.5	1	0			(ii)heterozygosity difference
745767	189	F	A	-	14	1	0	1			(ii)heterozygosity difference
745767	189	M	A	G	14	0.5	1	0			(ii)heterozygosity difference
745767	230	F	A	-	14	1	0	1			(ii)heterozygosity difference
745767	230	M	A	G	14	0.5	1	0			(ii)heterozygosity difference
745767	31	F	C	-	15	1	0	1			(ii)heterozygosity difference
745767	47	F	A	-	15	1	0	1			(ii)heterozygosity difference

745767	88	F	A	-	15	1	0	1			(ii)heterozygosity difference
773522	61	F	C	-	12	1	0	1	F		(ii)heterozygosity difference
773522	61	M	C	T	15	0.5	1	0			(ii)heterozygosity difference
775077	167	F	A	-	13	1	0	1	F		(ii)heterozygosity difference
775077	167	M	A	G	17	0.5	1	0			(ii)heterozygosity difference
801465	44	F	C	-	15	1	0	1	F		(ii)heterozygosity difference
801465	44	M	C	T	15	0.5	1	0			(ii)heterozygosity difference
801465	111	F	A	-	15	1	0	1			(ii)heterozygosity difference
801465	111	M	A	G	15	0.5	1	0			(ii)heterozygosity difference
801465	121	F	G	-	15	1	0	1			(ii)heterozygosity difference
801465	121	M	G	C	15	0.5	1	0			(ii)heterozygosity difference
801465	157	F	T	-	15	1	0	1			(ii)heterozygosity difference
801465	157	M	T	A	15	0.5	1	0			(ii)heterozygosity difference
801465	175	F	C	-	15	1	0	1			(ii)heterozygosity difference
801465	175	M	C	T	15	0.5	1	0			(ii)heterozygosity difference
801465	243	F	T	-	15	1	0	1			(ii)heterozygosity difference
801465	243	M	T	C	15	0.5	1	0			(ii)heterozygosity difference

805011	105	F	C	-	15	1	0	1	F		(ii)heterozygosity difference
805011	105	M	C	T	16	0.5	1	0			(ii)heterozygosity difference
815343	6	M	G	T	12	0.5	1	0	T	XY	(ii)heterozygosity difference
815343	263	M	C	T	12	0.5	1	0			(ii)heterozygosity difference
815343	6	F	G	-	16	1	0	1			(ii)heterozygosity difference
815343	263	F	C	-	16	1	0	1			(ii)heterozygosity difference
819489	217	M	G	A	13	0.5	1	0	T	XY	(ii)heterozygosity difference
819489	217	F	G	-	14	1	0	1			(ii)heterozygosity difference
828268	52	F	G	-	15	1	0	1	F		(ii)heterozygosity difference
828268	52	M	G	A	16	0.5	1	0			(ii)heterozygosity difference
838976	266	M	C	T	18	0.5	1	0	T	XY	(ii)heterozygosity difference
838976	280	M	T	C	18	0.5	1	0			(ii)heterozygosity difference
838976	266	F	C	-	21	1	0	1			(ii)heterozygosity difference
838976	280	F	T	-	21	1	0	1			(ii)heterozygosity difference
846766	17	F	T	-	14	1	0	1	F		(ii)heterozygosity difference
846766	265	F	T	-	14	1	0	1			(ii)heterozygosity difference
846766	17	M	T	A	20	0.5	1	0			(ii)heterozygosity difference

846766	265	M	T	G	20	0.5	1	0			(ii)heterozygosity difference
853624	130	F	T	-	15	1	0	1	T	XY	(ii)heterozygosity difference
853624	221	F	G	-	15	1	0	1			(ii)heterozygosity difference
853624	246	F	A	-	15	1	0	1			(ii)heterozygosity difference
853624	130	M	T	C	17	0.5	1	0			(ii)heterozygosity difference
853624	221	M	G	A	17	0.5	1	0			(ii)heterozygosity difference
853624	246	M	A	G	17	0.5	1	0			(ii)heterozygosity difference
879764	204	F	C	-	12	1	0	1	F		(ii)heterozygosity difference
879764	172	M	G	A	13	0.5	1	0			(ii)heterozygosity difference
879764	172	F	G	-	14	1	0	1			(ii)heterozygosity difference
879764	204	M	C	A	15	0.5	1	0			(ii)heterozygosity difference
881121	205	M	C	T	15	0.5	1	0	F		(ii)heterozygosity difference
881121	254	M	G	C	15	0.5	1	0			(ii)heterozygosity difference
881121	205	F	C	-	16	1	0	1			(ii)heterozygosity difference
881121	254	F	G	-	16	1	0	1			(ii)heterozygosity difference
887595	74	F	G	-	18	1	0	1	F		(ii)heterozygosity difference
887595	73	F	T	-	19	1	0	1			(ii)heterozygosity difference

887595	73	M	T	C	21	0.5	1	0			(ii)heterozygosity difference
887595	74	M	G	T	21	0.5	1	0			(ii)heterozygosity difference
891797	76	F	T	-	13	1	0	1	T	XY	(ii)heterozygosity difference
891797	76	M	T	C	14	0.5	1	0			(ii)heterozygosity difference
892228	19	M	G	A	15	0.5	1	0	T	XY	(ii)heterozygosity difference
892228	19	F	G	-	19	1	0	1			(ii)heterozygosity difference
892517	120	F	G	-	13	1	0	1	T	XY	(ii)heterozygosity difference
892517	120	M	G	A	13	0.5	1	0			(ii)heterozygosity difference
892519	190	M	C	A	18	0.5	1	0	F		(ii)heterozygosity difference
892519	190	F	C	-	19	1	0	1			(ii)heterozygosity difference
892519	198	F	C	-	19	1	0	1			(ii)heterozygosity difference
892519	198	M	C	G	19	0.5	1	0			(ii)heterozygosity difference
901415	230	F	C	-	13	1	0	1	F		(ii)heterozygosity difference
901415	189	M	A	G	14	0.5	1	0			(ii)heterozygosity difference
901415	189	F	A	-	17	1	0	1			(ii)heterozygosity difference
901415	230	M	C	A	18	0.5	1	0			(ii)heterozygosity difference
904391	175	F	T	-	12	1	0	1	F		(ii)heterozygosity difference

904391	175	M	T	A	18	0.5	1	0			(ii)heterozygosity difference
910249	51	F	C	-	15	1	0	1			(ii)heterozygosity difference
910249	51	M	C	G	15	0.5	1	0			(ii)heterozygosity difference
924532	54	F	C	-	20	1	0	1	T	XY	(ii)heterozygosity difference
924532	89	F	C	-	20	1	0	1			(ii)heterozygosity difference
924532	288	F	G	-	20	1	0	1			(ii)heterozygosity difference
924532	288	M	G	A	20	0.5	1	0			(ii)heterozygosity difference
924532	54	M	C	T	21	0.5	1	0			(ii)heterozygosity difference
924532	89	M	C	G	21	0.5	1	0			(ii)heterozygosity difference
933218	36	M	A	G	15	0.5	1	0	T	XY	(ii)heterozygosity difference
933218	36	F	A	-	16	1	0	1			(ii)heterozygosity difference
949676	91	F	G	-	15	1	0	1	T	XY	(ii)heterozygosity difference
949676	91	M	G	A	15	0.5	1	0			(ii)heterozygosity difference
1078436	203	M	C	T	14	0.5	1	0	F		(ii)heterozygosity difference
1078436	245	M	T	C	14	0.5	1	0			(ii)heterozygosity difference
1078436	203	F	C	-	16	1	0	1			(ii)heterozygosity difference
1078436	245	F	T	-	16	1	0	1			(ii)heterozygosity difference

1104005	37	F	G	-	13	1	0	1	F		(ii)heterozygosity difference
1104005	37	M	G	A	18	0.5	1	0			(ii)heterozygosity difference
1130720	58	M	G	A	14	0.5	1	0	T	XY	(ii)heterozygosity difference
1130720	103	M	G	C	14	0.5	1	0			(ii)heterozygosity difference
1130720	58	F	G	-	17	1	0	1			(ii)heterozygosity difference
1130720	103	F	G	-	17	1	0	1			(ii)heterozygosity difference
1214457	245	M	G	A	13	0.5	1	0	F		(ii)heterozygosity difference
1214457	19	F	G	-	15	1	0	1			(ii)heterozygosity difference
1214457	19	M	G	T	15	0.5	1	0			(ii)heterozygosity difference
1214457	130	F	T	-	15	1	0	1			(ii)heterozygosity difference
1214457	130	M	T	C	15	0.5	1	0			(ii)heterozygosity difference
1214457	194	F	T	-	15	1	0	1			(ii)heterozygosity difference
1214457	194	M	T	C	15	0.5	1	0			(ii)heterozygosity difference
1214457	245	F	G	-	15	1	0	1			(ii)heterozygosity difference
1267066	73	M	G	A	13	0.5	1	0	T	XY	(ii)heterozygosity difference
1267066	73	F	G	-	14	1	0	1			(ii)heterozygosity difference
1355306	35	F	G	-	12	1	0	1	F		(ii)heterozygosity difference

1355306	35	M	G	A	13	0.5	1	0			(ii)heterozygosity difference

Note:

Col, the nucleotide site within the catalog locus, reported using a zero-based offset (first nucleotide is enumerated as 0); Pop, we split samples into male and female: males were assigned to Pop M, females were assigned to Pop F; P.Nuc, the most frequent allele at this position in this population; Q.Nuc, the alternative allele; N, number of individuals sampled in this population at this site;P, frequency of most frequent allele; Obs.Het, the proportion of individuals that are heterozygotes in this population; Obs.Hom, the proportion of individuals that are homozygotes in this population; confirmed, validation of sex-linked markers, 'T' and 'F' indicate true and false, respectively.

Table S2 All details of sex-linked markers (continue)

Locus (CLocus_)	f.Count	m.Count	Confirmed (Tor F)	System	approach
2057836	0	14	F		(iii)sex-limited
2058893	0	11	F		(iii)sex-limited
2197675	0	11	T	XY	(iii)sex-limited
3469297	0	14	F		(iii)sex-limited
3469321	0	12	T	XY	(iii)sex-limited
3469575	0	11	F		(iii)sex-limited
3469625	0	17	F		(iii)sex-limited
3469669	0	11	T	XY	(iii)sex-limited
3469694	0	18	F		(iii)sex-limited
3469808	0	15	T	XY	(iii)sex-limited
3469918	0	16	T	XY	(iii)sex-limited
3469920	0	19	T	XY	(iii)sex-limited
3469959	0	11	F		(iii)sex-limited
3469970	0	12	F		(iii)sex-limited
3470013	0	17	F		(iii)sex-limited
3470311	0	15	T	XY	(iii)sex-limited
3470410	0	11	F		(iii)sex-limited
3470422	0	13	F		(iii)sex-limited
3470457	0	12	T	XY	(iii)sex-limited
3470463	0	17	F		(iii)sex-limited

3470595	0	13	F		(iii)sex-limited
3470647	0	11	F		(iii)sex-limited
3470692	0	15	F		(iii)sex-limited
3470769	0	14	F		(iii)sex-limited
3470801	0	18	T	XY	(iii)sex-limited
3470809	0	11	F		(iii)sex-limited
3470856	0	13	T	XY	(iii)sex-limited
3470873	0	19	T	XY	(iii)sex-limited
3470896	0	11	T	XY	(iii)sex-limited
3470911	0	13	F		(iii)sex-limited
3471090	0	12	F		(iii)sex-limited
3471133	0	17	F		(iii)sex-limited
3471141	0	17	T	XY	(iii)sex-limited
3471189	0	15	F		(iii)sex-limited
3471341	0	17	T	XY	(iii)sex-limited
3471535	0	12	T	XY	(iii)sex-limited
3471636	0	15	T	XY	(iii)sex-limited
3471660	0	17	F		(iii)sex-limited
3471668	0	11	T	XY	(iii)sex-limited
3471816	0	18	T	XY	(iii)sex-limited
3471858	0	18	T	XY	(iii)sex-limited
3471914	0	17	T	XY	(iii)sex-limited
3472026	0	11	F		(iii)sex-limited
3472044	0	12	F		(iii)sex-limited
3472060	0	12	T	XY	(iii)sex-limited

3472206	0	13	F		(iii)sex-limited
3472215	0	11	T	XY	(iii)sex-limited
3472227	0	14	T	XY	(iii)sex-limited
3472323	0	14	F		(iii)sex-limited
3472368	0	20	F		(iii)sex-limited
3472559	0	12	F		(iii)sex-limited
3472572	0	14	T	XY	(iii)sex-limited
3472589	0	13	T	XY	(iii)sex-limited
3472857	0	11	F		(iii)sex-limited
3472920	0	14	T	XY	(iii)sex-limited
3473082	0	14	T	XY	(iii)sex-limited
3473103	0	12	T	XY	(iii)sex-limited
3473179	0	14	T	XY	(iii)sex-limited
3473269	0	15	T	XY	(iii)sex-limited
3473485	0	12	T	XY	(iii)sex-limited
3473621	0	13	F		(iii)sex-limited
3473680	0	13	T	XY	(iii)sex-limited
3474203	0	11	T	XY	(iii)sex-limited
3474356	0	15	T	XY	(iii)sex-limited
3474373	0	17	T	XY	(iii)sex-limited
3474403	0	12	F		(iii)sex-limited
3474618	0	21	T	XY	(iii)sex-limited
3474734	0	16	F		(iii)sex-limited
3474845	0	15	T	XY	(iii)sex-limited
3474938	0	16	T	XY	(iii)sex-limited

3475064	0	15	T	XY	(iii)sex-limited
3475077	0	12	F		(iii)sex-limited
3475140	0	12	F		(iii)sex-limited
3475251	0	12	T	XY	(iii)sex-limited
3475522	0	15	T	XY	(iii)sex-limited
3475915	0	11	T	XY	(iii)sex-limited
3476039	0	13	T	XY	(iii)sex-limited
3476117	0	11	T	XY	(iii)sex-limited
3476218	0	11	F		(iii)sex-limited
3476278	0	16	T	XY	(iii)sex-limited
3476293	0	12	T	XY	(iii)sex-limited
3476436	0	12	F		(iii)sex-limited
3476517	0	11	F		(iii)sex-limited
3476810	0	11	T	XY	(iii)sex-limited
3476922	0	12	T	XY	(iii)sex-limited
3476929	0	11	F		(iii)sex-limited
3476955	0	14	F		(iii)sex-limited
3477015	0	13	F		(iii)sex-limited
3477054	0	15	T	XY	(iii)sex-limited
3477115	0	22	F		(iii)sex-limited
3477702	0	17	T	XY	(iii)sex-limited
3477784	0	13	F		(iii)sex-limited
3477897	0	14	T	XY	(iii)sex-limited
3477936	0	11	T	XY	(iii)sex-limited
3477966	0	14	F		(iii)sex-limited

3478060	0	19	T	XY	(iii)sex-limited
3478096	0	15	T	XY	(iii)sex-limited
3478319	0	11	F		(iii)sex-limited
3478370	0	16	F		(iii)sex-limited
3478374	0	13	F		(iii)sex-limited
3478416	0	17	F		(iii)sex-limited
3478453	0	11	T	XY	(iii)sex-limited
3478501	0	12	T	XY	(iii)sex-limited
3478563	0	13	F		(iii)sex-limited
3478623	0	19	T	XY	(iii)sex-limited
3478661	0	17	F		(iii)sex-limited
3478683	0	15	F		(iii)sex-limited
3478764	0	16	T	XY	(iii)sex-limited
3478772	0	14	T	XY	(iii)sex-limited
3478782	0	11	F		(iii)sex-limited
3478801	0	12	T	XY	(iii)sex-limited
3478811	0	13	T	XY	(iii)sex-limited
3478824	0	12	T	XY	(iii)sex-limited
3479001	0	11	T	XY	(iii)sex-limited
3479042	0	11	F		(iii)sex-limited
3479050	0	15	T	XY	(iii)sex-limited
3479312	0	11	T	XY	(iii)sex-limited
3479317	0	13	T	XY	(iii)sex-limited
3479331	0	15	F		(iii)sex-limited
3479351	0	13	F		(iii)sex-limited

3479506	0	15	F		(iii)sex-limited
3479530	0	12	F		(iii)sex-limited
3479694	0	15	T	XY	(iii)sex-limited
3479840	0	16	T	XY	(iii)sex-limited
3479922	0	16	T	XY	(iii)sex-limited
3480009	0	13	T	XY	(iii)sex-limited
3480105	0	15	T	XY	(iii)sex-limited
3480182	0	18	T	XY	(iii)sex-limited
3480462	0	13	F		(iii)sex-limited
3480497	0	13	T	XY	(iii)sex-limited
3480556	0	16	T	XY	(iii)sex-limited
3480646	0	14	T	XY	(iii)sex-limited
3480678	0	11	F		(iii)sex-limited
3480698	0	13	F		(iii)sex-limited
3480757	0	18	T	XY	(iii)sex-limited
3480869	0	16	F		(iii)sex-limited
3480982	0	12	F		(iii)sex-limited
3481194	0	11	F		(iii)sex-limited
3481205	0	13	T	XY	(iii)sex-limited
3481449	0	13	F		(iii)sex-limited
3481461	0	11	T	XY	(iii)sex-limited
3481826	0	14	T	XY	(iii)sex-limited
3481831	0	15	T	XY	(iii)sex-limited
3481852	0	11	F		(iii)sex-limited
3481881	0	16	T	XY	(iii)sex-limited

3481918	0	13	F		(iii)sex-limited
3482359	0	13	F		(iii)sex-limited
3482587	0	11	T	XY	(iii)sex-limited
3482600	0	13	F		(iii)sex-limited
3482650	0	13	F		(iii)sex-limited
3482802	0	18	T	XY	(iii)sex-limited
3482863	0	13	F		(iii)sex-limited
3483014	0	11	F		(iii)sex-limited
3483155	0	14	F		(iii)sex-limited
3483296	0	13	T	XY	(iii)sex-limited
3483297	0	12	T	XY	(iii)sex-limited
3483424	0	13	F		(iii)sex-limited
3483436	0	12	T	XY	(iii)sex-limited
3483555	0	15	T	XY	(iii)sex-limited
3483890	0	14	F		(iii)sex-limited
3484137	0	12	T	XY	(iii)sex-limited
3484189	0	11	F		(iii)sex-limited
3484268	0	16	T	XY	(iii)sex-limited
3484358	0	12	T	XY	(iii)sex-limited
3484449	0	13	F		(iii)sex-limited
3484463	0	12	T	XY	(iii)sex-limited
3484492	0	12	T	XY	(iii)sex-limited
3484961	0	20	T	XY	(iii)sex-limited
3485033	0	15	F		(iii)sex-limited
3485036	0	11	T	XY	(iii)sex-limited

3485117	0	13	F		(iii)sex-limited
3485183	0	14	T	XY	(iii)sex-limited
3485322	0	20	T	XY	(iii)sex-limited
3485472	0	16	F		(iii)sex-limited
3485641	0	20	T	XY	(iii)sex-limited
3485643	0	18	T	XY	(iii)sex-limited
3485738	0	15	F		(iii)sex-limited
3485805	0	11	F		(iii)sex-limited
3485870	0	11	T	XY	(iii)sex-limited
3485908	0	13	T	XY	(iii)sex-limited
3485929	0	13	F		(iii)sex-limited
3486034	0	11	F		(iii)sex-limited
3486495	0	17	F		(iii)sex-limited
3486586	0	11	T	XY	(iii)sex-limited
3486650	0	15	F		(iii)sex-limited
3486658	0	15	T	XY	(iii)sex-limited
3486698	0	14	T	XY	(iii)sex-limited
3486843	0	21	T	XY	(iii)sex-limited
3486911	0	12	T	XY	(iii)sex-limited
3486949	0	21	T	XY	(iii)sex-limited
3487069	0	11	T	XY	(iii)sex-limited
3487112	0	13	F		(iii)sex-limited
3487210	0	13	T	XY	(iii)sex-limited
3487585	0	14	T	XY	(iii)sex-limited
3487606	0	20	T	XY	(iii)sex-limited

3487616	0	14	T	XY	(iii)sex-limited
3487739	0	17	F		(iii)sex-limited
3487784	0	14	F		(iii)sex-limited
3487788	0	11	T	XY	(iii)sex-limited
3487901	0	14	T	XY	(iii)sex-limited
3487909	0	18	F		(iii)sex-limited
3488043	0	13	T	XY	(iii)sex-limited
3488068	0	12	F		(iii)sex-limited
3488109	0	18	T	XY	(iii)sex-limited
3488166	0	18	T	XY	(iii)sex-limited
3488464	0	18	F		(iii)sex-limited
3488469	0	15	T	XY	(iii)sex-limited
3488617	0	14	T	XY	(iii)sex-limited
3488711	0	12	F		(iii)sex-limited
3488779	0	12	T	XY	(iii)sex-limited
3488790	0	13	T	XY	(iii)sex-limited
3489021	0	21	T	XY	(iii)sex-limited
3489068	0	13	T	XY	(iii)sex-limited
3489086	0	11	F		(iii)sex-limited
3489269	0	18	T	XY	(iii)sex-limited
3489364	0	15	T	XY	(iii)sex-limited
3489432	0	12	T	XY	(iii)sex-limited
3489449	0	13	T	XY	(iii)sex-limited
3489520	0	14	F		(iii)sex-limited
3489539	0	14	F		(iii)sex-limited

3489629	0	13	T	XY	(iii)sex-limited
3489646	0	15	F		(iii)sex-limited
3489745	0	14	F		(iii)sex-limited
3489830	0	16	F		(iii)sex-limited
3489985	0	14	F		(iii)sex-limited
3490043	0	15	F		(iii)sex-limited
3490061	0	15	T	XY	(iii)sex-limited
3490245	0	19	T	XY	(iii)sex-limited
3490290	0	14	F		(iii)sex-limited
3490367	0	12	F		(iii)sex-limited
3490491	0	18	T	XY	(iii)sex-limited
3490534	0	11	F		(iii)sex-limited
3490584	0	18	F		(iii)sex-limited
3490677	0	17	F		(iii)sex-limited
3490782	0	12	F		(iii)sex-limited
3490783	0	19	T	XY	(iii)sex-limited
3490792	0	12	T	XY	(iii)sex-limited
3490944	0	16	T	XY	(iii)sex-limited
3491180	0	11	F		(iii)sex-limited
3491185	0	13	F		(iii)sex-limited
3491296	0	12	T	XY	(iii)sex-limited
3491326	0	14	T	XY	(iii)sex-limited
3491327	0	15	T	XY	(iii)sex-limited
3491376	0	16	T	XY	(iii)sex-limited
3491403	0	16	F		(iii)sex-limited

3491483	0	15	T	XY	(iii)sex-limited
3491570	0	12	T	XY	(iii)sex-limited
3491623	0	14	T	XY	(iii)sex-limited
3491733	0	12	T	XY	(iii)sex-limited
3491788	0	17	F		(iii)sex-limited
3491797	0	17	F		(iii)sex-limited
3491863	0	17	T	XY	(iii)sex-limited
3492006	0	17	T	XY	(iii)sex-limited
3492108	0	17	T	XY	(iii)sex-limited
3492374	0	13	F		(iii)sex-limited
3492419	0	16	F		(iii)sex-limited
3492461	0	11	T	XY	(iii)sex-limited
3492500	0	21	T	XY	(iii)sex-limited
3492596	0	15	T	XY	(iii)sex-limited
3492631	0	16	F		(iii)sex-limited
3492935	0	13	F		(iii)sex-limited
3493241	0	12	T	XY	(iii)sex-limited
3493322	0	22	T	XY	(iii)sex-limited
3493403	0	18	F		(iii)sex-limited
3493587	0	11	F		(iii)sex-limited
3493668	0	11	T	XY	(iii)sex-limited
3493789	0	21	T	XY	(iii)sex-limited
3493983	0	12	F		(iii)sex-limited
3494120	0	20	T	XY	(iii)sex-limited
3494264	0	11	T	XY	(iii)sex-limited

3494349	0	11	T	XY	(iii)sex-limited
3494519	0	19	T	XY	(iii)sex-limited
3494601	0	13	F		(iii)sex-limited
3494731	0	12	F		(iii)sex-limited
3494857	0	20	T	XY	(iii)sex-limited
3494876	0	13	F		(iii)sex-limited
3494912	0	19	T	XY	(iii)sex-limited
3494921	0	13	T	XY	(iii)sex-limited
3495247	0	11	F		(iii)sex-limited
3495503	0	12	F		(iii)sex-limited
3495647	0	15	F		(iii)sex-limited
3495676	0	17	T	XY	(iii)sex-limited
3495725	0	14	T	XY	(iii)sex-limited
3495734	0	16	F		(iii)sex-limited
3495959	0	11	T	XY	(iii)sex-limited
3496058	0	16	F		(iii)sex-limited
3496104	0	18	T	XY	(iii)sex-limited
3496452	0	14	T	XY	(iii)sex-limited
3496570	0	16	T	XY	(iii)sex-limited
3496614	0	12	T	XY	(iii)sex-limited
3496662	0	17	F		(iii)sex-limited
3496678	0	14	F		(iii)sex-limited
3496999	0	12	F		(iii)sex-limited
3497185	0	16	F		(iii)sex-limited
3497324	0	12	T	XY	(iii)sex-limited

3497491	0	12	F		(iii)sex-limited
3497499	0	11	F		(iii)sex-limited
3497559	0	13	T	XY	(iii)sex-limited
3497603	0	14	T	XY	(iii)sex-limited
3497639	0	17	T	XY	(iii)sex-limited
3497646	0	11	F		(iii)sex-limited
3497724	0	15	T	XY	(iii)sex-limited
3497730	0	12	F		(iii)sex-limited
3497768	0	16	T	XY	(iii)sex-limited
3497828	0	11	T	XY	(iii)sex-limited
3497937	0	13	F		(iii)sex-limited
3498001	0	13	T	XY	(iii)sex-limited
3498070	0	11	F		(iii)sex-limited
3498138	0	11	T	XY	(iii)sex-limited
3498254	0	13	T	XY	(iii)sex-limited
3498291	0	15	F		(iii)sex-limited
3498410	0	12	F		(iii)sex-limited
3498499	0	12	F		(iii)sex-limited
3498687	0	16	F		(iii)sex-limited
3498709	0	14	F		(iii)sex-limited
3498728	0	15	F		(iii)sex-limited
3498776	0	15	F		(iii)sex-limited
3498964	0	17	T	XY	(iii)sex-limited
3499208	0	12	F		(iii)sex-limited
3499279	0	13	F		(iii)sex-limited

3499294	0	11	F		(iii)sex-limited
3499455	0	11	F		(iii)sex-limited
3499466	0	16	T	XY	(iii)sex-limited
3499523	0	18	T	XY	(iii)sex-limited
3499576	0	11	F		(iii)sex-limited
3499634	0	22	T	XY	(iii)sex-limited
3499893	0	13	T	XY	(iii)sex-limited
3500011	0	13	T	XY	(iii)sex-limited
3500263	0	11	T	XY	(iii)sex-limited
3500350	0	14	T	XY	(iii)sex-limited
3500365	0	20	T	XY	(iii)sex-limited
3500482	0	11	F		(iii)sex-limited
3500496	0	11	F		(iii)sex-limited
3500529	0	15	T	XY	(iii)sex-limited
3500558	0	16	F		(iii)sex-limited
3500619	0	13	F		(iii)sex-limited
3500822	0	18	F		(iii)sex-limited
3500892	0	11	F		(iii)sex-limited
3501011	0	12	F		(iii)sex-limited
3501026	0	16	T	XY	(iii)sex-limited
3501073	0	13	T	XY	(iii)sex-limited
3501107	0	12	T	XY	(iii)sex-limited
3501440	0	16	T	XY	(iii)sex-limited
3501537	0	16	T	XY	(iii)sex-limited
3501560	0	14	T	XY	(iii)sex-limited

3501645	0	15	F		(iii)sex-limited
3501733	0	12	T	XY	(iii)sex-limited
3501771	0	11	T	XY	(iii)sex-limited
3501958	0	11	T	XY	(iii)sex-limited
3501973	0	12	F		(iii)sex-limited
3502028	0	20	T	XY	(iii)sex-limited
3502341	0	14	T	XY	(iii)sex-limited
3502485	0	14	T	XY	(iii)sex-limited
3502689	0	17	F		(iii)sex-limited
3502699	0	18	F		(iii)sex-limited
3502755	0	16	T	XY	(iii)sex-limited
3502802	0	20	F		(iii)sex-limited
3502851	0	15	T	XY	(iii)sex-limited
3502869	0	12	F		(iii)sex-limited
3502953	0	16	T	XY	(iii)sex-limited
3503004	0	14	F		(iii)sex-limited
3503095	0	15	T	XY	(iii)sex-limited
3503178	0	16	T	XY	(iii)sex-limited
3503231	0	13	F		(iii)sex-limited
3503266	0	11	T	XY	(iii)sex-limited
3503325	0	17	F		(iii)sex-limited
3503326	0	11	T	XY	(iii)sex-limited
3503522	0	16	F		(iii)sex-limited
3503547	0	11	T	XY	(iii)sex-limited
3503562	0	11	F		(iii)sex-limited

3503608	0	11	T	XY	(iii)sex-limited
3503627	0	14	T	XY	(iii)sex-limited
3503695	0	15	F		(iii)sex-limited
3503750	0	14	F		(iii)sex-limited
3503782	0	19	T	XY	(iii)sex-limited
3503859	0	15	F		(iii)sex-limited
3503955	0	19	T	XY	(iii)sex-limited
3503967	0	16	F		(iii)sex-limited
3503984	0	14	T	XY	(iii)sex-limited
3504016	0	19	F		(iii)sex-limited
3504400	0	13	T	XY	(iii)sex-limited
3504475	0	14	T	XY	(iii)sex-limited
3504545	0	13	T	XY	(iii)sex-limited
3504626	0	16	T	XY	(iii)sex-limited
3504632	0	12	F		(iii)sex-limited
3504723	0	16	T	XY	(iii)sex-limited
3504754	0	11	F		(iii)sex-limited
3504810	0	19	T	XY	(iii)sex-limited
3504825	0	13	F		(iii)sex-limited
3504879	0	17	T	XY	(iii)sex-limited
3504926	0	20	T	XY	(iii)sex-limited
3505013	0	21	T	XY	(iii)sex-limited
3505023	0	17	T	XY	(iii)sex-limited
3505027	0	13	F		(iii)sex-limited
3505106	0	14	T	XY	(iii)sex-limited

3505134	0	15	T	XY	(iii)sex-limited
3505187	0	16	T	XY	(iii)sex-limited
3505205	0	16	T	XY	(iii)sex-limited
3505315	0	18	F		(iii)sex-limited
3505465	0	11	T	XY	(iii)sex-limited
3505498	0	16	T	XY	(iii)sex-limited
3505525	0	18	T	XY	(iii)sex-limited
3505720	0	22	T	XY	(iii)sex-limited
3505828	0	13	F		(iii)sex-limited
3505871	0	13	T	XY	(iii)sex-limited
3505902	0	20	F		(iii)sex-limited
3505927	0	16	F		(iii)sex-limited
3506097	0	12	T	XY	(iii)sex-limited
3506125	0	12	F		(iii)sex-limited
3506175	0	13	T	XY	(iii)sex-limited
3506222	0	15	F		(iii)sex-limited
3506293	0	14	T	XY	(iii)sex-limited
3506553	0	19	T	XY	(iii)sex-limited
3506568	0	13	F		(iii)sex-limited
3506657	0	14	F		(iii)sex-limited
3506697	0	12	F		(iii)sex-limited
3506732	0	14	F		(iii)sex-limited
3507058	0	17	T	XY	(iii)sex-limited
3507208	0	12	T	XY	(iii)sex-limited
3507228	0	13	F		(iii)sex-limited

3507418	0	19	T	XY	(iii)sex-limited
3507459	0	12	F		(iii)sex-limited
3507539	0	19	T	XY	(iii)sex-limited
3507624	0	11	F		(iii)sex-limited
3507667	0	17	F		(iii)sex-limited
3507706	0	13	T	XY	(iii)sex-limited
3507945	0	12	T	XY	(iii)sex-limited
3508129	0	13	T	XY	(iii)sex-limited
3508189	0	14	T	XY	(iii)sex-limited
3508238	0	11	F		(iii)sex-limited
3508363	0	16	T	XY	(iii)sex-limited
3508423	0	16	F		(iii)sex-limited
3508498	0	17	T	XY	(iii)sex-limited
3508598	0	13	F		(iii)sex-limited
3508813	0	11	F		(iii)sex-limited
3509077	0	11	F		(iii)sex-limited
3509152	0	13	F		(iii)sex-limited
3509387	0	12	F		(iii)sex-limited
3509683	0	14	T	XY	(iii)sex-limited
3509732	0	11	T	XY	(iii)sex-limited
3509836	0	12	F		(iii)sex-limited
3509857	0	14	F		(iii)sex-limited
3509867	0	14	T	XY	(iii)sex-limited
3509875	0	13	T	XY	(iii)sex-limited
3509927	0	15	T	XY	(iii)sex-limited

3509950	0	14	T	XY	(iii)sex-limited
3510048	0	13	F		(iii)sex-limited
3510093	0	11	F		(iii)sex-limited
3510227	0	15	T	XY	(iii)sex-limited
3510380	0	16	T	XY	(iii)sex-limited
3510420	0	17	T	XY	(iii)sex-limited
3510439	0	21	T	XY	(iii)sex-limited
3510514	0	11	F		(iii)sex-limited
3510540	0	16	T	XY	(iii)sex-limited
3510544	0	12	F		(iii)sex-limited
3510632	0	11	T	XY	(iii)sex-limited
3510797	0	12	T	XY	(iii)sex-limited
3510841	0	11	F		(iii)sex-limited
3510989	0	12	F		(iii)sex-limited
3511023	0	19	T	XY	(iii)sex-limited
3511046	0	16	F		(iii)sex-limited
3511402	0	12	T	XY	(iii)sex-limited
3511455	0	11	T	XY	(iii)sex-limited
3511469	0	15	T	XY	(iii)sex-limited
3511586	0	11	F		(iii)sex-limited
3511639	0	18	T	XY	(iii)sex-limited
3511669	0	15	F		(iii)sex-limited
3511703	0	16	T	XY	(iii)sex-limited
3511765	0	16	F		(iii)sex-limited
3511826	0	15	F		(iii)sex-limited

3511887	0	15	F		(iii)sex-limited
3512009	0	13	T	XY	(iii)sex-limited
3512087	0	11	T	XY	(iii)sex-limited
3512119	0	15	T	XY	(iii)sex-limited
3512178	0	12	T	XY	(iii)sex-limited
3512251	0	14	T	XY	(iii)sex-limited
3512321	0	18	T	XY	(iii)sex-limited
3512453	0	14	T	XY	(iii)sex-limited
3512553	0	18	T	XY	(iii)sex-limited
3512768	0	12	F		(iii)sex-limited
3513001	0	16	T	XY	(iii)sex-limited
3513223	0	18	T	XY	(iii)sex-limited
3513232	0	16	T	XY	(iii)sex-limited
3513312	0	12	F		(iii)sex-limited
3513386	0	13	F		(iii)sex-limited
3513483	0	17	T	XY	(iii)sex-limited
3513632	0	11	T	XY	(iii)sex-limited
3513820	0	15	T	XY	(iii)sex-limited
3513880	0	15	T	XY	(iii)sex-limited
3513947	0	14	F		(iii)sex-limited
3514042	0	11	T	XY	(iii)sex-limited
3514162	0	13	T	XY	(iii)sex-limited
3514199	0	13	F		(iii)sex-limited
3514266	0	11	F		(iii)sex-limited
3514298	0	14	F		(iii)sex-limited

3514465	0	16	T	XY	(iii)sex-limited
3514647	0	12	T	XY	(iii)sex-limited
3514964	0	19	T	XY	(iii)sex-limited
3515143	0	15	T	XY	(iii)sex-limited
3515158	0	12	T	XY	(iii)sex-limited
3515306	0	15	F		(iii)sex-limited
3515398	0	12	T	XY	(iii)sex-limited
3515580	0	14	T	XY	(iii)sex-limited
3515589	0	19	T	XY	(iii)sex-limited
3515636	0	15	T	XY	(iii)sex-limited
3515665	0	14	T	XY	(iii)sex-limited
3515695	0	13	F		(iii)sex-limited
3515897	0	14	F		(iii)sex-limited
3516000	0	11	T	XY	(iii)sex-limited
3516116	0	14	T	XY	(iii)sex-limited
3516129	0	15	T	XY	(iii)sex-limited
3516160	0	13	T	XY	(iii)sex-limited
3516296	0	19	T	XY	(iii)sex-limited
3516363	0	12	F		(iii)sex-limited
3516464	0	12	T	XY	(iii)sex-limited
3516503	0	11	F		(iii)sex-limited
3516537	0	12	F		(iii)sex-limited
3516659	0	13	T	XY	(iii)sex-limited
3516752	0	12	T	XY	(iii)sex-limited
3516817	0	16	T	XY	(iii)sex-limited

3516828	0	11	F		(iii)sex-limited
3517150	0	16	T	XY	(iii)sex-limited
3517359	0	12	F		(iii)sex-limited
3517442	0	15	F		(iii)sex-limited
3517527	0	15	T	XY	(iii)sex-limited
3517681	0	15	T	XY	(iii)sex-limited
3518019	0	16	F		(iii)sex-limited
3518088	0	16	T	XY	(iii)sex-limited
3518145	0	17	T	XY	(iii)sex-limited
3518213	0	14	F		(iii)sex-limited
3518230	0	19	F		(iii)sex-limited
3518352	0	13	F		(iii)sex-limited
3518456	0	14	T	XY	(iii)sex-limited
3518494	0	11	F		(iii)sex-limited
3518541	0	12	F		(iii)sex-limited
3518593	0	11	T	XY	(iii)sex-limited
3518666	0	14	T	XY	(iii)sex-limited
3518678	0	18	T	XY	(iii)sex-limited
3518728	0	12	T	XY	(iii)sex-limited
3518844	0	15	T	XY	(iii)sex-limited
3519126	0	12	T	XY	(iii)sex-limited
3519273	0	13	F		(iii)sex-limited
3519281	0	11	T	XY	(iii)sex-limited
3519469	0	18	F		(iii)sex-limited
3519548	0	11	T	XY	(iii)sex-limited

3519557	0	14	T	XY	(iii)sex-limited
3519605	0	15	T	XY	(iii)sex-limited
3519636	0	13	T	XY	(iii)sex-limited
3519693	0	13	T	XY	(iii)sex-limited
3519704	0	13	T	XY	(iii)sex-limited
3519768	0	14	F		(iii)sex-limited
3519802	0	12	T	XY	(iii)sex-limited
3519855	0	16	T	XY	(iii)sex-limited
3519974	0	14	F		(iii)sex-limited
3520336	0	16	T	XY	(iii)sex-limited
3520390	0	12	T	XY	(iii)sex-limited
3520529	0	14	T	XY	(iii)sex-limited
3520539	0	13	T	XY	(iii)sex-limited
3520552	0	16	F		(iii)sex-limited
3520571	0	12	T	XY	(iii)sex-limited
3520645	0	14	T	XY	(iii)sex-limited
3520661	0	12	F		(iii)sex-limited
3520687	0	17	F		(iii)sex-limited
3520926	0	18	T	XY	(iii)sex-limited
3520930	0	13	F		(iii)sex-limited
3520951	0	14	T	XY	(iii)sex-limited
3520970	0	15	F		(iii)sex-limited
3521239	0	13	F		(iii)sex-limited
3521315	0	12	T	XY	(iii)sex-limited
3521374	0	11	T	XY	(iii)sex-limited

3521470	0	14	F		(iii)sex-limited
3521664	0	13	T	XY	(iii)sex-limited
3521848	0	14	T	XY	(iii)sex-limited
3522194	0	11	F		(iii)sex-limited
3522212	0	11	F		(iii)sex-limited
3522267	0	12	T	XY	(iii)sex-limited
3522412	0	14	T	XY	(iii)sex-limited
3522467	0	15	F		(iii)sex-limited
3522666	0	17	F		(iii)sex-limited
3522915	0	17	T	XY	(iii)sex-limited
3522918	0	16	F		(iii)sex-limited
3523118	0	14	F		(iii)sex-limited
3523329	0	12	T	XY	(iii)sex-limited
3523353	0	12	F		(iii)sex-limited
3523622	0	16	F		(iii)sex-limited
3523783	0	13	F		(iii)sex-limited
3523885	0	13	F		(iii)sex-limited
3524031	0	17	F		(iii)sex-limited
3524091	0	11	F		(iii)sex-limited
3524260	0	15	F		(iii)sex-limited
3524280	0	15	T	XY	(iii)sex-limited
3524325	0	17	F		(iii)sex-limited
3524372	0	12	T	XY	(iii)sex-limited
3524691	0	13	T	XY	(iii)sex-limited
3524728	0	14	T	XY	(iii)sex-limited

3524744	0	13	F		(iii)sex-limited
3525106	0	11	F		(iii)sex-limited
3525177	0	20	T	XY	(iii)sex-limited
3525199	0	14	T	XY	(iii)sex-limited
3525239	0	19	F		(iii)sex-limited
3525367	0	14	F		(iii)sex-limited
3525386	0	18	T	XY	(iii)sex-limited
3525513	0	16	T	XY	(iii)sex-limited
3525573	0	13	F		(iii)sex-limited
3525615	0	12	T	XY	(iii)sex-limited
3525734	0	11	F		(iii)sex-limited
3525898	0	13	F		(iii)sex-limited
3525996	0	14	T	XY	(iii)sex-limited
3526026	0	15	F		(iii)sex-limited
3526327	0	13	T	XY	(iii)sex-limited
3526373	0	14	T	XY	(iii)sex-limited
3526481	0	11	T	XY	(iii)sex-limited
3526491	0	11	T	XY	(iii)sex-limited
3526561	0	11	T	XY	(iii)sex-limited
3526598	0	11	T	XY	(iii)sex-limited
3528043	0	14	F		(iii)sex-limited
3529209	0	14	F		(iii)sex-limited
3529516	0	16	T	XY	(iii)sex-limited
3529518	0	14	F		(iii)sex-limited
3529783	0	11	T	XY	(iii)sex-limited

3530074	0	14	F		(iii)sex-limited
3530282	0	12	T	XY	(iii)sex-limited
3530677	0	17	F		(iii)sex-limited
3530888	0	14	T	XY	(iii)sex-limited
3532244	0	16	F		(iii)sex-limited
3532255	0	12	T	XY	(iii)sex-limited
3532294	0	11	T	XY	(iii)sex-limited
3532402	0	19	T	XY	(iii)sex-limited
3532977	0	12	T	XY	(iii)sex-limited
3534009	0	14	F		(iii)sex-limited
3534346	0	14	T	XY	(iii)sex-limited
3535140	0	13	T	XY	(iii)sex-limited
3535599	0	16	F		(iii)sex-limited
3535800	0	17	T	XY	(iii)sex-limited
3536059	0	15	F		(iii)sex-limited
3536122	0	16	T	XY	(iii)sex-limited
3536328	0	12	T	XY	(iii)sex-limited
3536551	0	11	F		(iii)sex-limited
3536578	0	12	T	XY	(iii)sex-limited
3536993	0	16	F		(iii)sex-limited
3537094	0	14	F		(iii)sex-limited
3537123	0	13	T	XY	(iii)sex-limited
3537517	0	16	T	XY	(iii)sex-limited
3537943	0	13	T	XY	(iii)sex-limited
3538518	0	15	T	XY	(iii)sex-limited

3538562	0	12	T	XY	(iii)sex-limited
3538646	0	12	F		(iii)sex-limited
3538842	0	11	T	XY	(iii)sex-limited
3539660	0	13	T	XY	(iii)sex-limited
3539891	0	14	T	XY	(iii)sex-limited
3539939	0	11	F		(iii)sex-limited
3540337	0	11	F		(iii)sex-limited
3540445	0	13	T	XY	(iii)sex-limited
3540475	0	18	T	XY	(iii)sex-limited
3540711	0	12	T	XY	(iii)sex-limited
3541007	0	11	T	XY	(iii)sex-limited
3541013	0	11	T	XY	(iii)sex-limited
3541164	0	11	T	XY	(iii)sex-limited
3541631	0	12	T	XY	(iii)sex-limited
3541739	0	13	T	XY	(iii)sex-limited
3542097	0	15	F		(iii)sex-limited
3542353	0	12	T	XY	(iii)sex-limited
3542391	0	19	T	XY	(iii)sex-limited
3542689	0	17	T	XY	(iii)sex-limited
3542807	0	18	F		(iii)sex-limited
3542867	0	15	T	XY	(iii)sex-limited
3542874	0	15	F		(iii)sex-limited
3542923	0	11	F		(iii)sex-limited
3543006	0	16	T	XY	(iii)sex-limited
3543098	0	16	F		(iii)sex-limited

3543131	0	15	F		(iii)sex-limited
3543361	0	11	F		(iii)sex-limited
3543788	0	15	T	XY	(iii)sex-limited
3543816	0	11	F		(iii)sex-limited
3543819	0	17	T	XY	(iii)sex-limited
3543831	0	11	F		(iii)sex-limited
3544070	0	15	F		(iii)sex-limited
3544121	0	15	T	XY	(iii)sex-limited
3544196	0	19	T	XY	(iii)sex-limited
3544246	0	11	F		(iii)sex-limited
3544268	0	13	T	XY	(iii)sex-limited
3545174	0	11	F		(iii)sex-limited
3545582	0	15	T	XY	(iii)sex-limited
3545707	0	11	T	XY	(iii)sex-limited
3545960	0	11	T	XY	(iii)sex-limited
3546693	0	11	F		(iii)sex-limited
3546842	0	11	F		(iii)sex-limited
3547201	0	11	F		(iii)sex-limited
3547289	0	12	T	XY	(iii)sex-limited
3547291	0	14	F		(iii)sex-limited
3547462	0	17	F		(iii)sex-limited
3548243	0	12	F		(iii)sex-limited
3548313	0	12	T	XY	(iii)sex-limited
3548573	0	12	T	XY	(iii)sex-limited
3548668	0	11	T	XY	(iii)sex-limited

3548739	0	12	T	XY	(iii)sex-limited
3548746	0	16	F		(iii)sex-limited
3548756	0	11	F		(iii)sex-limited
3549296	0	11	T	XY	(iii)sex-limited
3549565	0	13	F		(iii)sex-limited
3549734	0	12	T	XY	(iii)sex-limited
3549835	0	12	T	XY	(iii)sex-limited
3549848	0	15	T	XY	(iii)sex-limited
3550051	0	13	F		(iii)sex-limited
3550779	0	12	F		(iii)sex-limited
3551102	0	13	T	XY	(iii)sex-limited
3551428	0	15	T	XY	(iii)sex-limited
3551486	0	13	T	XY	(iii)sex-limited
3551558	0	11	T	XY	(iii)sex-limited
3551929	0	16	T	XY	(iii)sex-limited
3552314	0	13	T	XY	(iii)sex-limited
3552333	0	11	F		(iii)sex-limited
3552539	0	13	T	XY	(iii)sex-limited
3552617	0	13	T	XY	(iii)sex-limited
3552666	0	12	F		(iii)sex-limited
3552780	0	14	T	XY	(iii)sex-limited
3553203	0	15	F		(iii)sex-limited
3553417	0	12	F		(iii)sex-limited
3553555	0	15	T	XY	(iii)sex-limited
3553604	0	12	T	XY	(iii)sex-limited

3553710	0	11	T	XY	(iii)sex-limited
3554036	0	17	F		(iii)sex-limited
3554203	0	11	T	XY	(iii)sex-limited
3554864	0	11	T	XY	(iii)sex-limited
3554963	0	11	F		(iii)sex-limited
3554994	0	13	T	XY	(iii)sex-limited
3555486	0	14	F		(iii)sex-limited
3555943	0	11	F		(iii)sex-limited
3556051	0	15	T	XY	(iii)sex-limited
3557399	0	15	T	XY	(iii)sex-limited
3557449	0	11	T	XY	(iii)sex-limited
3558703	0	11	F		(iii)sex-limited
3558874	0	12	T	XY	(iii)sex-limited
3559125	0	12	T	XY	(iii)sex-limited
3559227	0	14	F		(iii)sex-limited
3559284	0	11	T	XY	(iii)sex-limited
3559334	0	11	F		(iii)sex-limited
3559508	0	15	T	XY	(iii)sex-limited
3560851	0	13	T	XY	(iii)sex-limited
3561984	0	12	T	XY	(iii)sex-limited
3562247	0	11	F		(iii)sex-limited
3562644	0	12	T	XY	(iii)sex-limited
3562690	0	17	T	XY	(iii)sex-limited
3563493	0	11	T	XY	(iii)sex-limited
3563743	0	11	T	XY	(iii)sex-limited

3563818	0	11	T	XY	(iii)sex-limited
3563956	0	13	F		(iii)sex-limited
3564205	0	16	T	XY	(iii)sex-limited
3564438	0	13	F		(iii)sex-limited
3565230	0	14	T	XY	(iii)sex-limited
3565301	0	14	T	XY	(iii)sex-limited
3565380	0	13	T	XY	(iii)sex-limited
3565670	0	11	F		(iii)sex-limited
3565695	0	12	F		(iii)sex-limited
3565699	0	13	F		(iii)sex-limited
3565836	0	11	F		(iii)sex-limited
3565871	0	11	T	XY	(iii)sex-limited
3566356	0	12	T	XY	(iii)sex-limited
3566387	0	11	F		(iii)sex-limited
3566485	0	13	F		(iii)sex-limited
3566698	0	15	T	XY	(iii)sex-limited
3567172	0	14	T	XY	(iii)sex-limited
3567490	0	11	T	XY	(iii)sex-limited
3567585	0	11	T	XY	(iii)sex-limited
3568092	0	13	T	XY	(iii)sex-limited
3569003	0	12	T	XY	(iii)sex-limited
3569450	0	15	T	XY	(iii)sex-limited
3569996	0	13	T	XY	(iii)sex-limited
3570134	0	12	T	XY	(iii)sex-limited
3570236	0	11	F		(iii)sex-limited

3570366	0	12	F		(iii)sex-limited
3570625	0	17	T	XY	(iii)sex-limited
3571023	0	12	F		(iii)sex-limited
3571421	0	13	F		(iii)sex-limited
3572013	0	14	F		(iii)sex-limited
3572106	0	13	F		(iii)sex-limited
3572288	0	11	F		(iii)sex-limited
3572297	0	11	F		(iii)sex-limited
3572380	0	14	T	XY	(iii)sex-limited
3572384	0	15	T	XY	(iii)sex-limited
3572413	0	15	T	XY	(iii)sex-limited
3572688	0	13	F		(iii)sex-limited
3573042	0	11	T	XY	(iii)sex-limited
3573315	0	14	T	XY	(iii)sex-limited
3573850	0	18	F		(iii)sex-limited
3573905	0	12	T	XY	(iii)sex-limited
3574084	0	13	F		(iii)sex-limited
3575283	0	13	T	XY	(iii)sex-limited
3575333	0	12	T	XY	(iii)sex-limited
3575524	0	14	F		(iii)sex-limited
3576898	0	11	T	XY	(iii)sex-limited
3577248	0	12	F		(iii)sex-limited
3577477	0	13	T	XY	(iii)sex-limited
3577925	0	13	F		(iii)sex-limited
3578339	0	17	T	XY	(iii)sex-limited

3578612	0	17	T	XY	(iii)sex-limited
3578800	0	12	F		(iii)sex-limited
3579787	0	12	T	XY	(iii)sex-limited
3579875	0	18	F		(iii)sex-limited
3580504	0	13	T	XY	(iii)sex-limited
3581051	0	16	F		(iii)sex-limited
3581142	0	11	T	XY	(iii)sex-limited
3581364	0	13	T	XY	(iii)sex-limited
3581401	0	14	F		(iii)sex-limited
3582767	0	12	T	XY	(iii)sex-limited
3584143	0	13	F		(iii)sex-limited
3584311	0	11	T	XY	(iii)sex-limited
3584455	0	12	F		(iii)sex-limited
3584616	0	16	F		(iii)sex-limited
3584698	0	18	T	XY	(iii)sex-limited
3584897	0	12	T	XY	(iii)sex-limited
3585032	0	11	F		(iii)sex-limited
3585147	0	13	T	XY	(iii)sex-limited
3585210	0	11	F		(iii)sex-limited
3585373	0	16	T	XY	(iii)sex-limited
3586317	0	11	T	XY	(iii)sex-limited
3586549	0	11	T	XY	(iii)sex-limited
3587651	0	12	F		(iii)sex-limited
3588675	0	13	T	XY	(iii)sex-limited
3590184	0	14	T	XY	(iii)sex-limited

3591810	0	11	F		(iii)sex-limited
3592553	0	13	T	XY	(iii)sex-limited
3593209	0	14	T	XY	(iii)sex-limited
3594501	0	11	T	XY	(iii)sex-limited
3595113	0	14	T	XY	(iii)sex-limited
3595273	0	12	F		(iii)sex-limited
3595569	0	13	T	XY	(iii)sex-limited
3596007	0	16	F		(iii)sex-limited
3596127	0	13	F		(iii)sex-limited
3597182	0	11	T	XY	(iii)sex-limited
3598525	0	12	T	XY	(iii)sex-limited
3601674	0	11	F		(iii)sex-limited
3603539	0	11	T	XY	(iii)sex-limited
3608759	0	13	F		(iii)sex-limited
3610274	0	11	F		(iii)sex-limited
3610563	0	14	T	XY	(iii)sex-limited
3611081	0	12	F		(iii)sex-limited
3611272	0	11	F		(iii)sex-limited
3615410	0	11	F		(iii)sex-limited
3617512	0	14	F		(iii)sex-limited
3618505	0	12	T	XY	(iii)sex-limited
3620994	0	16	F		(iii)sex-limited
3622561	0	12	F		(iii)sex-limited
3622577	0	11	F		(iii)sex-limited
3623796	0	13	F		(iii)sex-limited

3624884	0	14	F		(iii)sex-limited
3625105	0	11	T	XY	(iii)sex-limited
3629188	0	13	F		(iii)sex-limited
3629826	0	13	T	XY	(iii)sex-limited
3630110	0	14	T	XY	(iii)sex-limited
3631702	0	15	T	XY	(iii)sex-limited
3633014	0	13	T	XY	(iii)sex-limited
3634971	0	14	T	XY	(iii)sex-limited
3635738	0	13	T	XY	(iii)sex-limited
3636434	0	13	T	XY	(iii)sex-limited
3637598	0	12	F		(iii)sex-limited
3637789	0	12	F		(iii)sex-limited
3637925	0	12	F		(iii)sex-limited
3639606	0	12	T	XY	(iii)sex-limited
3640427	0	12	F		(iii)sex-limited
3647922	0	12	T	XY	(iii)sex-limited
3648599	0	12	F		(iii)sex-limited
3650574	0	11	F		(iii)sex-limited
3654610	0	11	F		(iii)sex-limited
3654617	0	11	F		(iii)sex-limited
3657088	0	12	F		(iii)sex-limited
3665839	0	13	T	XY	(iii)sex-limited
3666085	0	13	T	XY	(iii)sex-limited
3673940	0	14	F		(iii)sex-limited
3674563	0	14	T	XY	(iii)sex-limited

3674885	0	12	F		(iii)sex-limited
3681812	0	13	F		(iii)sex-limited
3688377	0	13	T	XY	(iii)sex-limited
3692848	0	12	F		(iii)sex-limited
3696564	0	12	T	XY	(iii)sex-limited
3703206	0	14	T	XY	(iii)sex-limited
3706677	0	18	T	XY	(iii)sex-limited
3718458	0	11	F		(iii)sex-limited
3720020	0	15	F		(iii)sex-limited
3722404	0	11	F		(iii)sex-limited
3724843	0	11	F		(iii)sex-limited
3726137	0	13	T	XY	(iii)sex-limited
3747592	0	12	F		(iii)sex-limited
3748106	0	11	T	XY	(iii)sex-limited
3760903	0	14	T	XY	(iii)sex-limited
3813167	0	11	T	XY	(iii)sex-limited
3861152	0	14	T	XY	(iii)sex-limited
3946270	0	17	T	XY	(iii)sex-limited
4011517	0	16	T	XY	(iii)sex-limited
4080251	0	14	T	XY	(iii)sex-limited
4090464	0	11	T	XY	(iii)sex-limited
4123548	0	12	F		(iii)sex-limited
4158334	0	17	T	XY	(iii)sex-limited
4180432	0	13	T	XY	(iii)sex-limited
4416225	0	12	T	XY	(iii)sex-limited

4523653	0	15	T	XY	(iii)sex-limited
4566633	0	20	T	XY	(iii)sex-limited
4601331	0	12	T	XY	(iii)sex-limited
4621005	0	13	T	XY	(iii)sex-limited
4635808	0	15	T	XY	(iii)sex-limited
4651769	0	11	T	XY	(iii)sex-limited
4660119	0	17	T	XY	(iii)sex-limited
4680887	0	11	T	XY	(iii)sex-limited
4701325	0	11	T	XY	(iii)sex-limited
4723076	0	12	T	XY	(iii)sex-limited
742720	10	0	T	ZW	(iii)sex-limited
807043	13	0	T	ZW	(iii)sex-limited
938798	10	0	F		(iii)sex-limited
1000516	10	0	T	ZW	(iii)sex-limited
1051324	10	0	T	ZW	(iii)sex-limited
1146063	10	0	F		(iii)sex-limited
1148713	13	0	F		(iii)sex-limited
1245343	10	0	F		(iii)sex-limited
1261469	10	0	F		(iii)sex-limited
1298316	12	0	F		(iii)sex-limited
1303857	10	0	T	ZW	(iii)sex-limited
1340598	10	0	F		(iii)sex-limited
1353382	14	0	F		(iii)sex-limited
1387449	10	0	F		(iii)sex-limited
1501190	10	0	F		(iii)sex-limited

1623757	11	0	F		(iii)sex-limited
1636926	10	0	T	ZW	(iii)sex-limited
1732515	10	0	F		(iii)sex-limited
1734989	11	0	T	ZW	(iii)sex-limited
1774506	11	0	F		(iii)sex-limited
2014425	10	0	F		(iii)sex-limited

Note:

locus, locus ID; f.Count, the number of individuals that sex-limited loci occurrence in female population; m.Count, the number of individuals that sex-limited loci occurrence in male population; confirmed, validation of sex-linked markers, 'T' and 'F' indicate true and false, respectively.

Table S3 Comparing the sex-linked loci with the scaffold 1345 of *N. parkeri*

GBS-tags	Subject id	e-value	q. start	q. end
CLocus_465977	scaffold1345	2.01E-42	950	729
CLocus_3552780	scaffold1345	9.42E-36	6718	6864
CLocus_3518145	scaffold1345	9.42E-36	6864	6718
CLocus_3595569	scaffold1345	9.49E-36	22575	22850
CLocus_3500011	scaffold1345	4.45E-24	40623	40518
CLocus_712545	scaffold1345	2.05E-27	47998	48090
CLocus_52943	scaffold1345	1.62E-48	48108	47967
CLocus_3597182	scaffold1345	9.63E-21	62690	62751
CLocus_3523329	scaffold1345	6.35E-92	82367	82637
CLocus_3482587	scaffold1345	6.35E-92	82637	82367
CLocus_3538562	scaffold1345	3.37E-40	104598	104480
CLocus_3544268	scaffold1345	4.45E-24	110529	110449
CLocus_3496452	scaffold1345	1.18E-59	127788	127602
CLocus_70112	scaffold1345	8.79E-46	127788	127640
CLocus_3487069	scaffold1345	1.11E-44	134998	134830
CLocus_3580504	scaffold1345	7.39E-27	142623	142515
CLocus_3501733	scaffold1345	7.42E-27	142623	142515
CLocus_3507706	scaffold1345	3.29E-55	149062	148933
CLocus_3509732	scaffold1345	1.19E-49	151635	151778
CLocus_3505023	scaffold1345	2.46E-81	164970	165258
CLocus_3519693	scaffold1345	2.46E-81	165258	164970
CLocus_3542867	scaffold1345	2.58E-46	176865	176656

CLocus_3485641	scaffold1345	1.24E-24	189971	190100
CLocus_3522267	scaffold1345	3.29E-55	191943	191762
CLocus_3503608	scaffold1345	1.90E-82	191943	191689
CLocus_35863	scaffold1345	9.42E-36	200063	200154
CLocus_3488779	scaffold1345	4.38E-34	200305	200412
CLocus_3575333	scaffold1345	5.75E-23	204552	204612
CLocus_3586549	scaffold1345	1.60E-23	213660	213753
CLocus_3518666	scaffold1345	9.42E-36	216990	217231
CLocus_3484492	scaffold1345	3.13E-30	217108	217273
CLocus_3501073	scaffold1345	1.46E-38	217219	216990
CLocus_3513880	scaffold1345	1.19E-49	217274	217012
CLocus_3491623	scaffold1345	1.53E-53	225809	225587
CLocus_3474373	scaffold1345	2.10E-27	226134	226014
CLocus_838976	scaffold1345	2.62E-36	253317	253204
CLocus_3502851	scaffold1345	4.38E-34	257242	257154
CLocus_3475522	scaffold1345	1.11E-34	285217	285453
CLocus_3487585	scaffold1345	1.19E-49	286386	286643
CLocus_3519704	scaffold1345	3.34E-45	286386	286643
CLocus_3495959	scaffold1345	1.19E-49	286386	286553
CLocus_3473179	scaffold1345	3.23E-70	286392	286610
CLocus_4090464	scaffold1345	7.08E-57	286398	286643
CLocus_3760903	scaffold1345	1.91E-62	286553	286386
CLocus_4180432	scaffold1345	4.29E-69	286610	286392
CLocus_3491296	scaffold1345	5.32E-78	286643	286378
CLocus_3494349	scaffold1345	3.34E-45	286643	286386

CLocus_3603539	scaffold1345	9.13E-76	286643	286386
CLocus_3639606	scaffold1345	1.69E-93	286979	287276
CLocus_3503984	scaffold1345	3.23E-70	287229	286979
CLocus_1267066	scaffold1345	1.57E-38	292862	292727
CLocus_3480556	scaffold1345	1.52E-58	302355	302632
CLocus_3511469	scaffold1345	1.52E-58	302632	302355
CLocus_3549835	scaffold1345	9.36E-41	309568	309698
CLocus_3501440	scaffold1345	3.32E-50	325991	326270
CLocus_3511703	scaffold1345	9.16E-56	325991	326270
CLocus_3519855	scaffold1345	4.01E-59	325991	326270
CLocus_3505134	scaffold1345	5.59E-43	325991	326270
CLocus_3480497	scaffold1345	8.76E-46	325991	326270
CLocus_455017	scaffold1345	1.98E-52	325991	326270
CLocus_3507418	scaffold1345	2.58E-46	326001	326270
CLocus_3813167	scaffold1345	1.80E-67	326001	326249
CLocus_24490	scaffold1345	7.03E-67	326001	326270
CLocus_3480182	scaffold1345	2.00E-47	326004	326270
CLocus_3634971	scaffold1345	1.54E-48	326006	326270
CLocus_3484268	scaffold1345	5.47E-58	326013	326281
CLocus_3476293	scaffold1345	1.19E-54	326013	326270
CLocus_73728	scaffold1345	4.23E-69	326013	326287
CLocus_1130720	scaffold1345	9.36E-41	326013	326270
CLocus_3504926	scaffold1345	7.23E-42	326017	326270
CLocus_3497768	scaffold1345	1.17E-64	326025	326270
CLocus_3515636	scaffold1345	2.38E-61	326025	326270

CLocus_3485036	scaffold1345	1.97E-57	326026	326270
CLocus_199491	scaffold1345	6.70E-57	326028	326270
CLocus_3503627	scaffold1345	1.48E-38	326037	326270
CLocus_3503326	scaffold1345	1.49E-33	326037	326270
CLocus_3514964	scaffold1345	7.13E-52	326040	326281
CLocus_3543819	scaffold1345	4.23E-59	326040	326270
CLocus_3497724	scaffold1345	2.58E-46	326040	326270
CLocus_3553555	scaffold1345	7.13E-52	326040	326270
CLocus_3557399	scaffold1345	3.29E-55	326040	326270
CLocus_3578612	scaffold1345	1.54E-48	326042	326270
CLocus_3551929	scaffold1345	3.37E-40	326042	326270
CLocus_3530888	scaffold1345	2.44E-36	326042	326270
CLocus_3565380	scaffold1345	1.46E-48	326042	326270
CLocus_3521315	scaffold1345	6.45E-62	326042	326270
CLocus_3519281	scaffold1345	1.89E-37	326042	326270
CLocus_263452	scaffold1345	6.93E-47	326042	326270
CLocus_3503955	scaffold1345	1.19E-49	326043	326270
CLocus_3483297	scaffold1345	5.04E-58	326051	326270
CLocus_3543006	scaffold1345	1.57E-38	326168	326270
CLocus_3471914	scaffold1345	7.29E-37	326171	326270
CLocus_566624	scaffold1345	1.26E-44	326171	326281
CLocus_3482802	scaffold1345	4.42E-29	326179	326270
CLocus_3518844	scaffold1345	9.46E-36	326179	326270
CLocus_3492500	scaffold1345	1.24E-39	326270	326040
CLocus_3487606	scaffold1345	3.13E-55	326270	326043

CLocus_3478623	scaffold1345	1.19E-49	326270	326043
CLocus_3480757	scaffold1345	1.54E-48	326270	326006
CLocus_3513223	scaffold1345	4.26E-54	326270	325991
CLocus_3525386	scaffold1345	5.63E-38	326270	326172
CLocus_3510420	scaffold1345	1.17E-64	326270	326025
CLocus_3522915	scaffold1345	3.31E-55	326270	326040
CLocus_3505187	scaffold1345	3.37E-40	326270	326042
CLocus_3513001	scaffold1345	2.00E-47	326270	326004
CLocus_3516129	scaffold1345	3.43E-35	326270	326171
CLocus_3544121	scaffold1345	8.76E-46	326270	325991
CLocus_3569450	scaffold1345	1.43E-63	326270	326013
CLocus_4635808	scaffold1345	1.55E-43	326270	325991
CLocus_3480646	scaffold1345	7.47E-37	326270	326171
CLocus_3501560	scaffold1345	9.16E-56	326270	325991
CLocus_3573315	scaffold1345	2.04E-32	326270	326171
CLocus_3539660	scaffold1345	2.00E-52	326270	326040
CLocus_3510797	scaffold1345	1.49E-33	326270	326037
CLocus_4723076	scaffold1345	2.43E-51	326270	326005
CLocus_3476117	scaffold1345	7.18E-47	326270	326042
CLocus_3513632	scaffold1345	1.89E-67	326270	326001
CLocus_3541013	scaffold1345	2.05E-27	326270	326179
CLocus_3567490	scaffold1345	6.45E-62	326270	326042
CLocus_4680887	scaffold1345	3.45E-30	326270	326179
CLocus_34389	scaffold1345	1.55E-53	326270	326001
CLocus_3544196	scaffold1345	1.24E-54	326281	326042

CLocus_3478764	scaffold1345	2.00E-47	326281	326039
CLocus_3520645	scaffold1345	9.03E-66	326281	326024
CLocus_285497	scaffold1345	1.98E-52	326281	326001
CLocus_3506097	scaffold1345	2.07E-22	327541	327462
CLocus_3503266	scaffold1345	1.58E-33	330251	330352
CLocus_3541007	scaffold1345	1.58E-33	330352	330251
CLocus_3488469	scaffold1345	1.17E-64	352341	352061
CLocus_3674563	scaffold1345	5.55E-48	374009	374232
CLocus_3563743	scaffold1345	5.55E-48	374232	374009
CLocus_3520926	scaffold1345	2.57E-51	376035	376175
CLocus_3505205	scaffold1345	2.55E-56	376035	376178
CLocus_891797	scaffold1345	7.29E-37	376077	376185
CLocus_3490061	scaffold1345	2.00E-47	376172	376035
CLocus_3485643	scaffold1345	2.57E-51	376175	376035
CLocus_3503095	scaffold1345	2.55E-56	376178	376035
CLocus_3486698	scaffold1345	7.08E-57	379754	379899
CLocus_3541739	scaffold1345	5.44E-63	379755	379899
CLocus_3473103	scaffold1345	2.53E-61	379755	379899
CLocus_3501958	scaffold1345	2.53E-61	379755	379899
CLocus_3512321	scaffold1345	5.51E-58	379756	379899
CLocus_3471535	scaffold1345	1.97E-57	379757	379899
CLocus_3472060	scaffold1345	7.23E-42	379776	379899
CLocus_3490792	scaffold1345	9.29E-46	379776	379879
CLocus_80479	scaffold1345	3.06E-55	379776	379899
CLocus_3540711	scaffold1345	9.29E-46	379879	379776

CLocus_3696564	scaffold1345	5.10E-53	379899	379776
CLocus_3501026	scaffold1345	1.14E-84	381144	380948
CLocus_3470856	scaffold1345	4.11E-79	381146	380948
CLocus_3554203	scaffold1345	8.58E-31	394131	394294
CLocus_3543788	scaffold1345	4.45E-24	394323	394256
CLocus_3509950	scaffold1345	9.16E-56	400406	400652
CLocus_3502028	scaffold1345	9.16E-56	400652	400406
CLocus_3517681	scaffold1345	7.34E-32	430264	430360
CLocus_3480009	scaffold1345	2.05E-27	435919	435841
CLocus_3556051	scaffold1345	1.22E-34	459355	459481
CLocus_3504879	scaffold1345	9.36E-41	460311	460444
CLocus_3581364	scaffold1345	5.59E-43	460328	460436
CLocus_3502341	scaffold1345	9.36E-41	460444	460311

Note: we aligned 574 confirmed sex-linked loci to the scaffold 1345 of Nanorana parkeri via Blast; Query id, sex linked markes from GBS-tags; Subject id, scaffold of *N. parkeri*; q. start, the start site of the GBS-tag on the scaffold 1345; q. end, the end site of the GBS-tag on the scaffold 1345; red font indicated that GBS-tags were verified by PCR.

Table S4 Confirmed sex-linked markers Mapped on *N. parkeri* Dmrt1

Query id	Subject id	e-value	q. start	q. end
CLocus_3474203	NW_017306666.1	5.59E-40	7155	7297
CLocus_3512178	NW_017306666.1	5.59E-40	7297	7155
CLocus_70112	NW_017306666.1	5.25E-45	7645	7793
CLocus_3541631	NW_017306666.1	1.24E-36	10811	10914
CLocus_3523329	NW_017306666.1	2.50E-23	12967	13080
CLocus_3485036	NW_017306666.1	1.56E-35	12967	13050
CLocus_3482587	NW_017306666.1	2.50E-23	13080	12967
CLocus_838976	NW_017306666.1	1.56E-35	15299	15413
CLocus_3518678	NW_017306666.1	1.59E-25	15311	15466
CLocus_52943	NW_017306666.1	3.49E-47	15413	15281
CLocus_61677	NW_017306666.1	7.33E-29	15466	15311
CLocus_3575333	NW_017306666.1	3.43E-22	15573	15724
CLocus_386683	NW_017306666.1	9.55E-23	15785	15932
CLocus_3549835	NW_017306666.1	2.64E-28	15860	15726
CLocus_3581364	NW_017306666.1	7.33E-29	27389	27532
CLocus_3491623	NW_017306666.1	9.09E-58	31672	31896
CLocus_3475522	NW_017306666.1	5.05E-45	31683	31946
CLocus_3480009	NW_017306666.1	5.70E-25	31853	31931
CLocus_3496452	NW_017306666.1	1.95E-59	31931	31790
CLocus_3490783	NW_017306666.1	3.29E-52	31965	32108
CLocus_3584698	NW_017306666.1	3.29E-52	32108	31965
CLocus_3538562	NW_017306666.1	1.22E-31	49418	49494

CLocus_3507706	NW_017306666.1	1.18E-51	54105	53978
CLocus_3563743	NW_017306666.1	1.56E-35	54282	54426
CLocus_3674563	NW_017306666.1	1.56E-35	54426	54282
CLocus_3594501	NW_017306666.1	1.83E-104	56615	56898
CLocus_3530282	NW_017306666.1	1.97E-54	58726	58849

Note: we aligned 574 confirmed sex-linked loci to the *N. parkeri* Dmrt1 gene via Blast; Query id, sex linked markes from GBS-tags; Subject id, Dmrt1 scaffold of *N. parkeri*; q. start, the start site of the GBS-tag on Dmrt1 gene; q. end, the end site of the GBS-tag on Dmrt1 gene; red font indicated that the locus CLocus_70112 (QS18) was verified by PCR.
